# Comparison of Healthy and Dandruff Scalp Microbiome Reveals the Role of Commensals in Scalp Health

**DOI:** 10.3389/fcimb.2018.00346

**Published:** 2018-10-04

**Authors:** Rituja Saxena, Parul Mittal, Cecile Clavaud, Darshan B. Dhakan, Prashant Hegde, Mahesh M. Veeranagaiah, Subarna Saha, Luc Souverain, Nita Roy, Lionel Breton, Namita Misra, Vineet K. Sharma

**Affiliations:** ^1^Department of Biological Sciences, Indian Institute of Science Education and Research Bhopal, Bhopal, India; ^2^L'Oréal Research & Innovation, Aulnay-sous-Bois, France; ^3^L'Oréal India Pvt. Ltd., Bengaluru, India

**Keywords:** scalp microbiome, metagenomics, dandruff, biotin, B-vitamins, *Malassezia*

## Abstract

Several scalp microbiome studies from different populations have revealed the association of dandruff with bacterial and fungal dysbiosis. However, the functional role of scalp microbiota in scalp disorders and health remains scarcely explored. Here, we examined the bacterial and fungal diversity of the scalp microbiome and their potential functional role in the healthy and dandruff scalp of 140 Indian women. *Propionibacterium acnes* and *Staphylococcus epidermidis* emerged as the core bacterial species, where the former was associated with a healthy scalp and the latter with dandruff scalp. Along with the commonly occurring *Malassezia* species (*M. restricta* and *M. globosa*) on the scalp, a strikingly high association of dandruff with yet uncharacterized *Malassezia* species was observed in the core mycobiome. Functional analysis showed that the fungal microbiome was enriched in pathways majorly implicated in cell-host adhesion in the dandruff scalp, while the bacterial microbiome showed a conspicuous enrichment of pathways related to the synthesis and metabolism of amino acids, biotin, and other B-vitamins, which are reported as essential nutrients for hair growth. A systematic measurement of scalp clinical and physiological parameters was also carried out, which showed significant correlations with the microbiome and their associated functional pathways. The results point toward a new potential role of bacterial commensals in maintaining the scalp nutrient homoeostasis and highlights an important and yet unknown role of the scalp microbiome, similar to the gut microbiome. This study, therefore, provides new perspectives on the better understanding of the pathophysiology of dandruff.

## Introduction

The scalp surface provides a distinct microenvironment to the microbes, primarily arising from the host physiological conditions, which include the sebum content, moisture, pH, and topography (Oh et al., 2014; Xu et al., 2016). Microbial communities confer advantageous survival on host surfaces, such as the cutaneous sites through different regulatory processes, including biofilm formation and quorum sensing (Brandwein et al., 2016). Thus, it is intended that the metabolic exchanges between the scalp surface and the microbiome typically support the growth of microbial biofilms in a symbiotic, commensal, or pathogenic form (Oh et al., 2014; Brandwein et al., 2016).

A dysbiosis in the cutaneous microbiome has been reported in the case of dandruff, seborrheic dermatitis, and atopic dermatitis (Park et al., 2012; Clavaud et al., 2013; Chng et al., 2016; Tanaka et al., 2016). However, the scalp microbiome studies conducted so far have focused only on determining the taxonomic composition and diversity variations in the bacterial and fungal communities in different populations. These studies have revealed *Malassezia*, and *Propionibacterium* and *Staphylococcus* as the prominent fungal and bacterial genera, respectively, and have also accentuated the individual or population-specific compositional variations (Yatsunenko et al., 2012; Clavaud et al., 2013; Saxena and Sharma, 2016; Soares et al., 2016). The association of the prominent fungal and bacterial genera, more particularly the association of *Malassezia* yeasts, with various scalp conditions has been reported frequently (Brüggemann et al., 2004; Deangelis et al., 2007; Otto, 2009; Clavaud et al., 2013; Magiatis et al., 2013; Grice and Dawson, 2017; Zhu et al., 2017). However, their pathogenic role and potential as a causal agent remains unclear.

The last decade has been instrumental in establishing the functional impact of the human gut microbiome on health, and recently, the role of skin microbiome on the skin health through various mechanisms, such as immune response modulation of the host and protection against skin pathogens, is also becoming apparent (Byrd et al., 2018; Eyerich et al., 2018; Meisel et al., 2018). Skin microbiome interacts with host keratinocytes and innate immune system leading to a stimulated secretion of the antimicrobial peptides, free fatty acids, cytokines, and chemokines, thus being beneficial for the host (Gallo and Nakatsuji, 2011; Meisel et al., 2018). However, the functional role of scalp microbiome, specifically the bacteriome, is yet unknown, which is essential for a better understanding of its influence on scalp health, and the pathophysiology of scalp-related disorders.

In this study, we carried out a large-scale amplicon and shotgun metagenome-based study of an Indian cohort consisting of 140 women of similar (20–45 years) age-range. To gain comparative insights on the potential role of scalp microbiome for the host, with respect to the commonly occurring dandruff condition, an equal number of healthy and dandruff scalp samples were included (Piérard-Franchimont et al., 2006; Misery et al., 2013). Since, the variations in the skin-physiology are reported to play an essential role in shaping the skin microbiome, the scalp clinical and physiological parameters, such as sebum level, trans-epidermal water loss (TEWL), and hydration level were also recorded, in addition to the dandruff severity grading (Xu et al., 2016).

The present study provides new perspectives for understanding the roles of bacteria and fungi in dandruff scalp. Here, we report a prominent association of healthy scalp microbiota with the synthesis of biotin and other vitamins, which are less represented in the dandruff scalp.

## Materials and methods

### Subject recruitment

The research protocol was approved by the Independent Ethics Committee for Evaluation of Protocols for Clinical Research, CLINICOM, Bengaluru, India (Study number ARC/COSB/1444) and was conducted in accordance with the principles expressed in the World Medical Association Declaration of Helsinki. A written informed consent was obtained from all subjects prior to any study-related procedures.

A total of 184 female volunteers were screened for the study, out of which 70 subjects with healthy and 70 subjects with dandruff scalp were enrolled with the association of MS Clinical research (Bengaluru, India). The volunteers were healthy females, aged 20–45 years (Table S1). All participants provided information regarding medical history and daily habits. Volunteers were non-smokers, free from any cutaneous diseases, did not consume or apply antibiotics or systemic antifungals for 1 month prior to sampling, did not use anti-hair-loss treatment at least for 3 months prior to sampling, and did not use anti-dandruff shampoos and hair-related products (such as for dyeing, bleaching, permanent waving, straightening, etc.) on scalp and hair for at least 3 weeks prior to sampling. Volunteers were asked to use a bland or neutral shampoo (L'Oréal India Pvt. Ltd.) two times a week for a period of 4 weeks prior to the beginning of the study to standardize the scalp condition.

### Dandruff grading and clinical evaluation of scalp physiological parameters

Dandruff level was scored according to a grading scale as previously described (Clavaud et al., 2013). Briefly, the scalp was divided into eight sections and adherent dandruff scores ranging from 0 to 5 were assigned to each area by comparing with reference pictures (Figure S1). The values were averaged to obtain a final score. The subjects with healthy scalp exhibited dandruff scores ≤1 and subjects with dandruff scalp had a dandruff score ≥2.5 (Table S2).

Scalp physiological parameters were also measured using appropriate devices and protocols. Sebumeter® (SM815, Courage & Khazaka, Germany) was used to measure the sebum level of the scalp following manufacturer's instructions. VapoMeter® (Delfin Technologies, Finland) was used to measure the TEWL (trans-epidermal water loss) that measures the integrity of barrier function of the scalp by following the manufacturer's instructions (De Paepe et al., 2005). All the measurements were performed in triplicates and values presenting coefficient of variation <15% were considered as relevant. Corneometer® (CK Electronic GmbH, Germany) measurement was performed on the scalp surface to check the hydration levels following the manufacturer's instructions. The above measurements were performed on a shaved trimmed mini-area before the shampoo wash at the investigational site by a dedicated operator.

In total, the following eight clinical or physiological parameters were recorded for each subject: (i) adherent dandruff score (ADS), (ii) total dandruff score (TDS) i.e., adherent and non-adherent, (iii) age, (iv) sebum level, (v) hydration, (vi) TEWL, (vii) itching, and (viii) erythema.

### Sampling of the scalp microbiome

Volunteers were advised not to wash their scalp for 2 days prior to the sampling procedure. Samples from the scalp (vertex or crown of the head) were obtained. For dandruff subjects, the area presenting a dandruff score of ≥2.5 on the vertex was selected. Sampling was conducted as previously described with minor modifications (Clavaud et al., 2013). Briefly, a sterile cotton swab soaked in a solution containing collection solution (0.15M NaCl and 0.1% Tween 20) was rubbed onto the scalp surface (between the hairs) under a zig-zag pattern, to cover a total surface of 4 cm^2^ in a non-overlapping manner. At the end of the procedure, the head of each swab was cut from the handle and placed into a tube containing 5 ml of collection buffer. As described previously (Clavaud et al., 2013), swabs were stored at 4°C and processed for DNA isolation within 24 h. In addition, a few sterile cotton swabs (as negative controls) were cut from the handle and placed in the collection buffer, and further processed using identical procedure.

### Fungal and bacterial metagenomic DNA extraction

Genomic DNA was extracted from the swab samples using DNeasy blood and tissue kit (Qiagen, MD, USA) as per the manufacturer's instructions with minor modifications for separate extraction of bacterial and fungal genomic DNA. The DNA extraction method was validated by genomic DNA extraction of a range of bacterial and fungal species tested separately, which gave a similar amount of DNA per cell (Table S3). Two identical samples of 2 ml each were generated from one collection tube. The microbial cell suspension from each tube was pelleted by centrifugation at 10,000 g for 30 min, at 4°C. For bacterial DNA extraction, the cells were re-suspended in 180 ml of lysis buffer (20 mM Tris-HCl, 2 mM EDTA, 1.2% TritonX100 (w/v); pH 8.0) and incubated for 30 min at 37°C. Further 25 μl of proteinase K and 200 μl buffer AL (Qiagen, MD, USA) were added to the mixture and incubated for 30 min at 56°C.

For fungal DNA extraction, the cells were re-suspended in 600 ml of lysis buffer (1M Sorbitol, 100 mM EDTA, 14 mM β-mercaptoethanol) and incubated with Zymolyase-T20 (200 U) for 30 min at 30°C. The resulting spheroplasts were centrifuged for 10 min at 300 × g and the supernatant was discarded. The spheroplasts were re-suspended in 180 μl of Buffer ATL and incubated with 20 μl of proteinase K (Qiagen, MD, USA) for 15 min at 56°C.

The remaining steps were performed according to the manufacturer's protocol and the extracted DNA was stored at −20°C. DNA concentration was measured using Qubit ds DNA HS kit on Qubit 2.0 fluorometer (Life technologies, Carlsbard, CA, USA).

### PCR amplification and sequencing

Equal concentration of bacterial and fungal DNA (~1 ng) was used for PCR amplification of bacterial 16S rRNA V3 hypervariable region and fungal ITS1 region (see Supplementary Methods for details of primers and protocols). After evaluating the amplified products on 2% w/v agarose gel, the products were purified using Ampure XP kit (Beckman Coulter, Brea, CA USA). Amplicon libraries were prepared using primers for V3 and ITS1 regions by following the Illumina 16S metagenomic library preparation guide.

Among the 140 subjects, 14 subjects with healthy scalp and 14 with dandruff scalp (28 subjects) were selected for shotgun metagenome sequencing of their bacterial and fungal DNA, based on the minimal DNA concentration (>0.2 ng/μl) required to carry out the library preparation for shotgun sequencing. The metagenomic libraries of the fungal and bacterial metagenomic DNA samples were prepared using Illumina Nextera XT sample preparation kit (Illumina Inc., USA).

Both the amplicon and shotgun metagenome libraries were evaluated on 2100 Bioanalyzer using DNA1000 kit for amplicon, and High Sensitivity DNA kit for metagenome (Agilent Technologies, Santa Clara, CA, USA) to estimate the library size. The libraries were further quantified on a Qubit 2.0 fluorometer using Qubit dsDNA HS kit (Life technologies, USA) and by qPCR using KAPA SYBR FAST qPCR Master mix and Illumina standards and primer premix (KAPA Biosystems, Wilmington, MA, USA) following the Illumina suggested protocol. Libraries in equal concentrations were loaded on Illumina NextSeq 500 platform using NextSeq 500/550 v2 sequencing reagent kit (Illumina Inc., USA) and 150 bp paired-end sequencing was performed for both types of libraries at the Next-Generation Sequencing (NGS) Facility, IISER Bhopal, India.

### Assignment of bacterial 16S rRNA (V3) and fungal ITS1 amplicon reads

The raw sequence data (Table S4) was subjected to quality trimming and ambiguity filtering using NGSQC toolkit and the paired-end reads were assembled for each amplicon sequence using FLASH (Magoc and Salzberg, 2011; Patel and Jain, 2012). Quality filtration of fastq reads was carried out using NGSQC toolkit and all the reads with 80% of bases ≥Q30 quality scores were selected for further analysis. Primers from the reads were removed using Cutadapt (Martin, 2011) and reads without the primer sequences were discarded. Clustering was carried out using closed-reference OTU picking and *de novo* OTU picking protocol of QIIME v1.9 (Caporaso et al., 2010) at ≥97% identity.

The custom ITS1 database (described in Supplementary Methods) and Greengenes database v13_5 were used as a reference for fungal and bacterial taxonomic assignment, respectively (Desantis et al., 2006). For the taxonomic assignment of *de novo* OTUs, sequences were aligned against the respective databases using BLAT, and the assignment was performed using Lowest Common Ancestor (LCA) algorithm (Kent, 2002; Desantis et al., 2006). The negative control samples showed a high abundance of fungal genus *Pachysolen* and bacterial genus *Actinotalea*, which are commonly found in environments such as air and soil, and not associated with the skin or scalp (Yi et al., 2007; Liu et al., 2012). Hence, the OTUs from these genera were excluded from the analysis.

The alpha diversity was calculated from amplicon reads using the metrics Shannon index and observed species after rarefying from 100 sequences at a step size of 6,000 for V3 as well as for ITS1 amplicons using QIIME v1.9. Beta diversity was analyzed by measuring the weighted UniFrac distances for the bacterial population. UniFrac distances were not calculated for fungal samples, due to the highly variable nature of ITS1 sequences, which makes them difficult in interpreting informative and meaningful phylogenetic analysis (Lindahl et al., 2013; Leung et al., 2016).

### Read processing for the whole metagenome sequencing data sets

The raw reads obtained from fungal and bacterial samples (Table S5) were filtered using NGSQC toolkit, and the reads with 60% bases above Q25 were considered for the analysis (Patel and Jain, 2012). The high-quality data obtained was further filtered to remove the bacterial and human contaminant reads. For fungal metagenome, the bacterial contaminant reads were removed by alignment against the bacterial reference genomes retrieved from NCBI, and the human contaminant reads were removed using BMTagger (Rotmistrovsky and Agarwala, 2011). The remaining fungal reads from each sample were assembled independently into contigs using SOAPdenovo at a k-mer size of 75 bp. The contigs with length ≥300 bp were selected and the genes were predicted from scaffolds using AUGUSTUS (Stanke and Morgenstern, 2005; Luo et al., 2012; Oh et al., 2014).

For bacterial metagenome, the human and fungal contaminant reads were removed by aligning the sequences using BLAT against human HG19 assembly and custom fungal genome database, respectively (Rosenbloom et al., 2014). These fungal genes and genomes were downloaded from Aspergillus database, FungiDB (release-30), Fungal Genome Initiative-Broad Institute, Fungi Ensembl, Saccharomyces Genome Database, Candida Genome Database, and NCBI to construct a custom fungal genome database (Kent, 2002; Cherry et al., 2011; Stajich et al., 2011; Cerqueira et al., 2013; Kersey et al., 2016; Skrzypek et al., 2017). If any fungal gene sequences were not available in these databases, the gene sequences were extracted from the genome sequences based on the gtf information using SAMtools (Li et al., 2009). The high-quality reads were assembled at a k-mer length of 47 (the k-mer length was estimated using kmerGenie, Chikhi and Medvedev, 2013) using SOAPdenovo, and the genes were predicted from the assembled contigs using MetaGeneMark (Zhu et al., 2010; Luo et al., 2012).

To ensure maximum identification of genes from the fungal genomes found in our dataset, the non-redundant fungal gene set consisting of 27,937 genes from assembled metagenomes were combined with CDS obtained from 303 fungal genomes retrieved from the Ensembl genomes database, and a total of 207,763 genes were used as a reference gene catalog (Qin et al., 2012). This comprehensive fungal gene catalog was used for mapping the metagenomic reads from each sample and to identify their respective genes. The bacterial gene catalog was generated by using CD-HIT, which consisted of 19,729,749 bacterial genes. In total, 81,395 fungal and 587,400 bacterial, non-redundant genes were identified in the dataset (see Supplementary Methods for details on gene quantification).

### Taxonomic assignment of reads from metagenomic data

The fungal reference genomes were retrieved from National Centre for Biotechnology Information (NCBI). The bacterial and archaeal genomes were retrieved from NCBI and Ensembl database. The metagenomic reads were aligned to the reference fungal and bacterial genomes and the mapped reads were considered for further analysis (Altschul et al., 1990; Langmead and Salzberg, 2012).

### Assignment of genes to KEGG orthologous groups

The KEGG v60 was updated by retrieving new sequences for KO IDs from the KEGG server. The bacterial and fungal genes were annotated by alignment against KEGG and eggNOG v4.0 databases (Zhao et al., 2011; Kanehisa et al., 2014; Powell et al., 2014; Buchfink et al., 2015). Protein sequences were assigned to eggNOG and KO orthologous groups based on the highest scoring hit containing at least one HSP (highest-scoring segment pair) above 60 bits and E-value ≤10^−6^. For bacteria, an additional parameter of identity >50% with ≥60% coverage or aligned length >300 was used. The relative abundance of each KO was calculated by adding up the abundance of genes mapping to the same KO ID, which was then used to calculate the relative abundance of KO ID in each sample. A similar approach was used to calculate the relative abundance of eggNOGs in each sample. In total, 4,064 and 3,532 KO IDs were obtained from the combined fungal and bacterial gene catalog, respectively. Pathway abundance was calculated by adding the relative abundance of each KO ID belonging to a particular pathway.

### Statistical analysis

All statistical analyses were performed using R software. The species (from amplicon analysis) and KEGG pathway composition were compared between the healthy and dandruff group using Wilcoxon test to identify significant (*p* ≤ 0.05) variations. All the comparisons were performed pairwise for each group. Differentially abundant pathways were identified using Wilcoxon test with FDR adj. *p*-values and Reporter Features Algorithm, which uses the Odds Ratio and the Wilcoxon *p-*values of each KO ID.

The Spearman's Rank Correlation Coefficients with FDR adj. *p*-value were calculated to correlate clinical parameters with species and KEGG pathways. Species with ≥1% proportion in at least 20 samples were selected for the correlation analysis. All the clinical parameters, except age and erythema, were chosen for all correlation analyses. Age and erythema were excluded, since the age-range was narrow and did not show significant correlations in any of the correlation analyses, and erythema remained stable during the study. Hierarchical clustering algorithm was used for clustering the highly-correlated pathways and species in the samples.

### Data availability

The high-throughput sequence data that support the findings of this study have been deposited in the National Centre for Biotechnology Information (NCBI) BioProject database under the project number PRJNA415710 and will be made publicly available on publication or on request at the peer review.

## Results

### Species diversity and taxonomic composition of healthy and dandruff scalp

**Figure 1:**
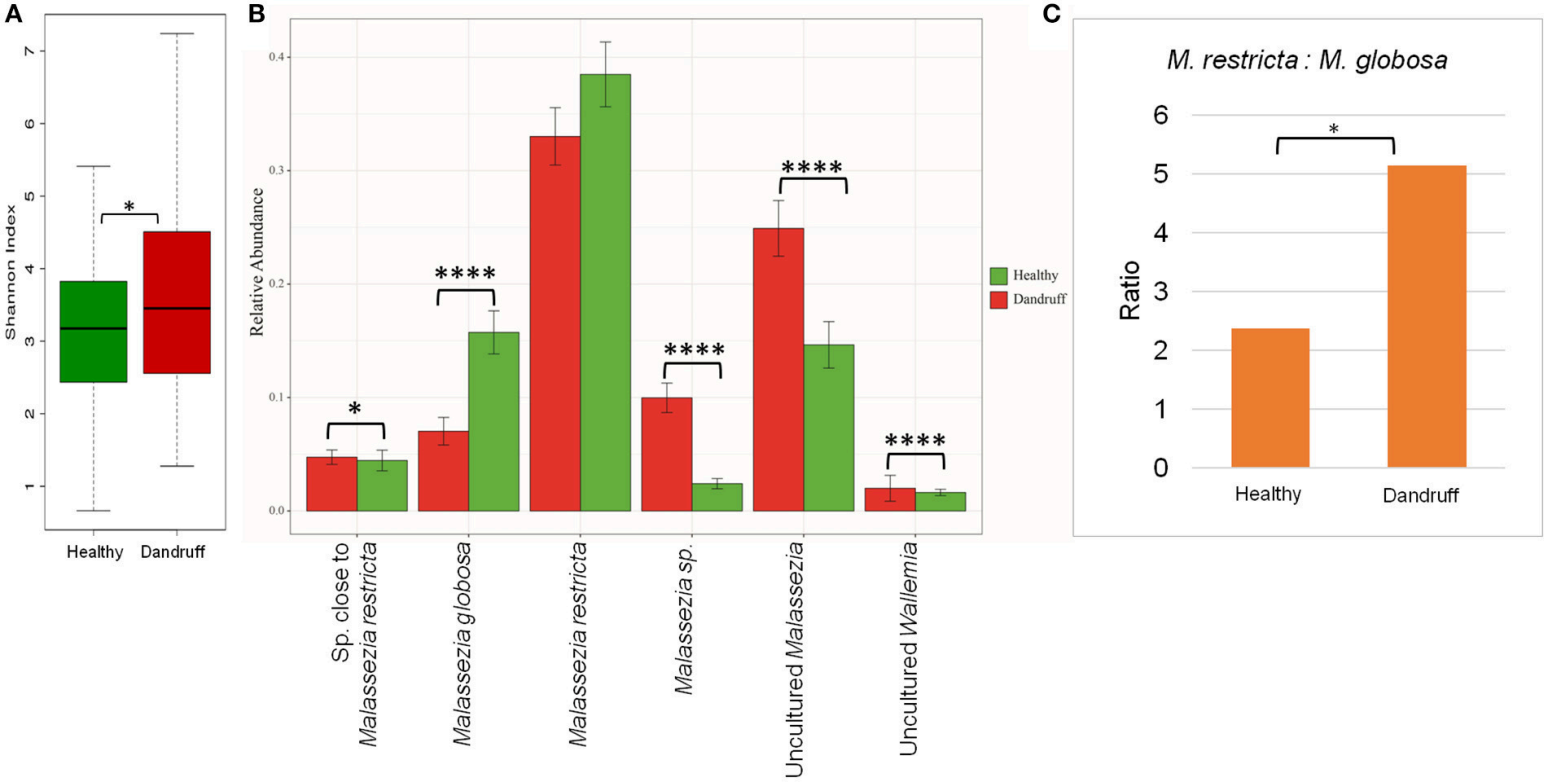
Species diversity and taxonomic composition of fungal microbiome in healthy and dandruff scalp. **(A)** Shannon diversity index for fungal population observed in healthy and dandruff scalp (*p ≤ 0.05). **(B)** Mean relative abundance of top five fungal species in healthy and dandruff scalp (****p ≤ 0.0001, *p ≤ 0.05, Wilcoxon test). **(C)** Differences in the ratio of *Malassezia restricta* and *Malassezia globosa* in the healthy and dandruff scalp (*p ≤ 0.05).

The scalp microbiome from 70 subjects with healthy (H) scalp and 70 with dandruff (D) scalp was established by determining the fungal and bacterial diversity through amplicon sequencing. Although a notable inter-individual variation in the taxonomic composition was observed, the prominent fungal species belonged to the genera *Malassezia* (>70%)*, Aspergillus, Cladosporium, Wallemia*, and *Alternaria* (Figures S2A,B). It was observed that the alpha-diversity (Shannon diversity index) for the fungal population was significantly lower (*p* ≤ 0.001) in the healthy scalp compared to the dandruff scalp (Figure 1A). At the species level, a high abundance of *M. restricta* and *M. globosa*, was observed in most of the samples. The abundance of *M. globosa* was significantly higher (*p* ≤ 0.0001) in the healthy scalp (16.23%) as compared to the dandruff scalp (6.41%) (Figure 1B). The abundance of *M. restricta* did not show a significant difference (*p* ≥ 0.05) between the healthy (38.42%) and dandruff (33%) scalp. However, the ratio of *M. restricta* to *M. globosa* was observed to be significantly higher (*p* = 0.004) in the dandruff scalp (Figure 1C).

A large part of the ITS1 amplicons (>40% of the total reads) were assigned to OTUs of uncharacterized *Malassezia* species. One of these OTUs (~20% of the total reads) belonged to uncultured species of *Malassezia* (>95% identity with Uncultured *Malassezia*, Genbank ID - KC785585.1) and others belonged to unknown *Malassezia* species, of which, six OTUs showed an identity of ≥85% with *M. restricta*, and were assigned to a subgroup of *Malassezia*. Therefore, the uncharacterized *Malassezia* sequences formed three subgroups: (1) uncultured *Malassezia* (~20% of total reads), (2) *Malassezia* sp. (~7% of total reads), and (3) species close to *M. restricta* (~4% of total reads). The uncultured *Malassezia* was observed to be significantly abundant (*p* ≤ 0.0001) in dandruff scalp (25.26%) than in the healthy scalp (14.44%) (Figure 1B). Unknown species of *Malassezia* and species close to *M. restricta* were also significantly (*p* ≤ 0.01) higher in the dandruff scalp compared to the healthy scalp. A high proportion of uncharacterized *Malassezia* (>37% of the total reads) was also observed in a recent study carried out in a Brazilian population (Soares et al., 2016). These findings suggest a key role of the hitherto uncharacterized *Malassezia* sp. in the pathophysiology of dandruff, and indicates that their characterization, at the strain level, would be imperative to gain a better understanding of the incidence of *Malassezia* sp. in dandruff and for the development of population-specific biomarkers for scalp-related disorders (Gaitanis et al., 2009).

Additionally, shotgun metagenomic analysis of the scalp samples was also carried out. The results confirmed *M. restricta* and *M. globosa* to be the most abundant fungi in both healthy and dandruff scalp. Further, *M. furfur, M. obtusa, M. pachydermatis*, and *M. caprae* were also identified in lower abundance(Figures S3A,B.)

**Figure 2:**
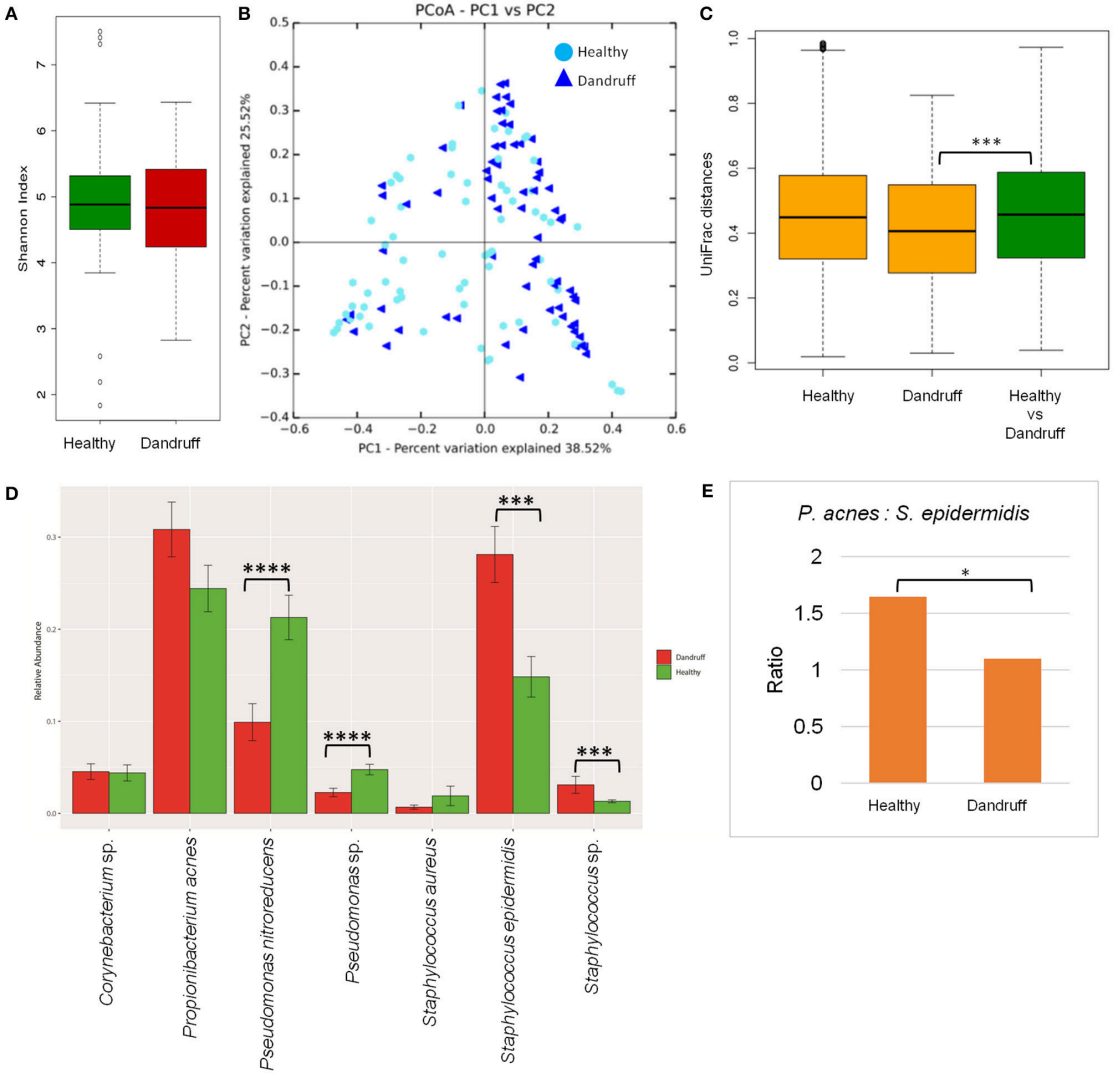
Species diversity and taxonomic composition of bacterial microbiome in healthy and dandruff scalp**. (A)** Shannon diversity index for bacterial population observed in healthy and dandruff scalp. **(B,C)** Unweighted UniFrac distances calculated for the bacterial population in healthy and dandruff scalp (***p ≤ 0.001). Orange bars represent the intra-sample distances within healthy and within dandruff groups, whereas green bar represents inter-sample distances between the healthy and dandruff groups. **(D)** Mean relative abundance of top five bacterial species in healthy and dandruff scalp (****p ≤ 0.0001, ***p ≤ 0.001, Wilcoxon test). **(E)** Differences in the ratio of *Propionibacterium acnes* and *Staphylococcus epidermidis* in the healthy and dandruff scalp (*p ≤ 0.05).

Bacterial species belonging to the genera *Propionibacterium, Staphylococcus, Corynebacterium, Bacillus*, and *Pseudomonas* were abundant in healthy and dandruff scalp (Figures S2C,D). Among the bacterial population, the alpha-diversity did not show a significant difference between the healthy and dandruff scalp (Figure 2A). Weighted UniFrac distances for the bacterial population showed higher distances within the healthy scalp than the dandruff scalp (Figure 2B.) Adonis test of association was also performed between the healthy and dandruff groups, which further supported the differences in healthy and dandruff groups (*R*^2^ = 0.04 and *p* = 0.002). The R^2^ value indicates that only 4% of the variation in distances is explained by this grouping. The UniFrac distances also showed a significant difference between the healthy and dandruff scalp, suggesting a difference in the community structure (Figure 2C). The abundance of *S. epidermidis* was observed to be significantly higher (*p* = 0.0002) in dandruff (28.1%) than in healthy (14.8%) scalp (Figure 2D). The abundance of *P. acnes* did not vary significantly (*p* ≥ 0.05) between dandruff (30.83%) and healthy (24.42%) scalp, however, the ratio of *P. acnes* to *S. epidermidis* was significantly higher (*p* = 0.01) in the healthy scalp compared to the dandruff scalp (Figure 2E). Further, *Pseudomonas nitroreducens* was also significantly abundant (*p* ≤ 0.0001) in healthy (21.2%) than in dandruff (9.9%) scalp. *Pseudomonas* spp. other than *P. nitroreducens* were also observed to be associated with scalp in previous studies (Clavaud et al., 2013).

The bacterial taxonomic assignment of metagenomic reads confirmed the predominance of *Staphylococcus* and *Propionibacterium* spp., as observed from the amplicon analysis (Figures S3C,D). In addition to *S. epidermidis*, which was also observed to be abundant using the amplicon analysis, the metagenomics dataset also allowed us to identify other *Staphylococcus* spp. such as *S. caprae, S capitis*, and *S. hominis* were also identified in the metagenomic analysis. Similar observations were also made in Chinese individuals, where an increase in the abundance of *S. epidermidis, S. capitis*, and *S. caprae* was observed in the dandruff scalp compared to the healthy scalp (Wang et al., 2015). *Propionibacterium* sp. CC003-HC2 was observed to be highly abundant, which is an HMP (Human Microbiome Project) isolate and has shown a close identity with *P. acnes* in a previous study (Mcdowell et al., 2013). A higher abundance of *Propionibacterium* spp. and a lower abundance of *Staphylococcus* spp. in healthy group compared to the dandruff group was confirmed using the metagenomic data (Figure S4.)

Determination of a core microbiome is important to understand the stable and consistent microbial population in the microbiome associated with any environment. The most stringent definition of any OTU to be a member of the core microbiome requires its presence in all the subjects (100%) sampled for the study (Huse et al., 2012). Since, there is a wide variation in the physiological parameters of the scalp across all the subjects in this study, the core OTUs which are dominant in some samples can be rare or absent in others. Therefore, a less stringent criteria of being ≥1% abundance in at least 80% of the samples (including the healthy and dandruff subjects) was used to define the core microbiome of the scalp. It was observed that *P. acnes* and *S. epidermidis* formed the bacterial core, whereas, *M. restricta* and *M. globosa* along with the *Malassezia* subgroups (1, 2, and 3) constituted the fungal core of the scalp. The significantly high abundance of uncharacterized *Malassezia* subgroups in dandruff scalp compared to the healthy scalp, and their presence across the individuals is a noteworthy observation, which could be specific to the Indian population.

### Correlation of microbial species with host clinical and physiological parameters

Previous studies have suggested a prominent role of host-associated physiological factors in the manifestation of dandruff (Xu et al., 2016; Pouradier et al., 2017). A systematic investigation of the host physiological parameters was carried out to study the association of host physiology with dandruff and with the scalp microbiome (Table S2). A significant positive correlation (FDR adj. *p* ≤ 0.05) was observed between the dandruff scores, itching and TEWL (Figure S5). The hydration and sebum levels also correlated positively with each other. Further, TEWL was observed to show a negative correlation with these two parameters. Comparison of the physiological parameters was also performed between the healthy and dandruff groups(Figure S6). This suggested the levels of dandruff scores and itching to be significantly higher in the dandruff scalp (*p* ≤ 0.0001, Wilcoxon test). Also, TEWL was observed to be significantly higher in the dandruff scalp compared to the healthy scalp (*p* = 0.035). It is known that alterations in the sebum level causes perturbations in the cutaneous barrier quality of the scalp, which leads to an increased TEWL in dandruff scalp than in healthy scalp and further perturbs the scalp barrier function (Turner et al., 2012). The correlation results from this study also suggest similar findings in the scalp-associated host physiological parameters of the Indian subjects.

**Figure 3:**
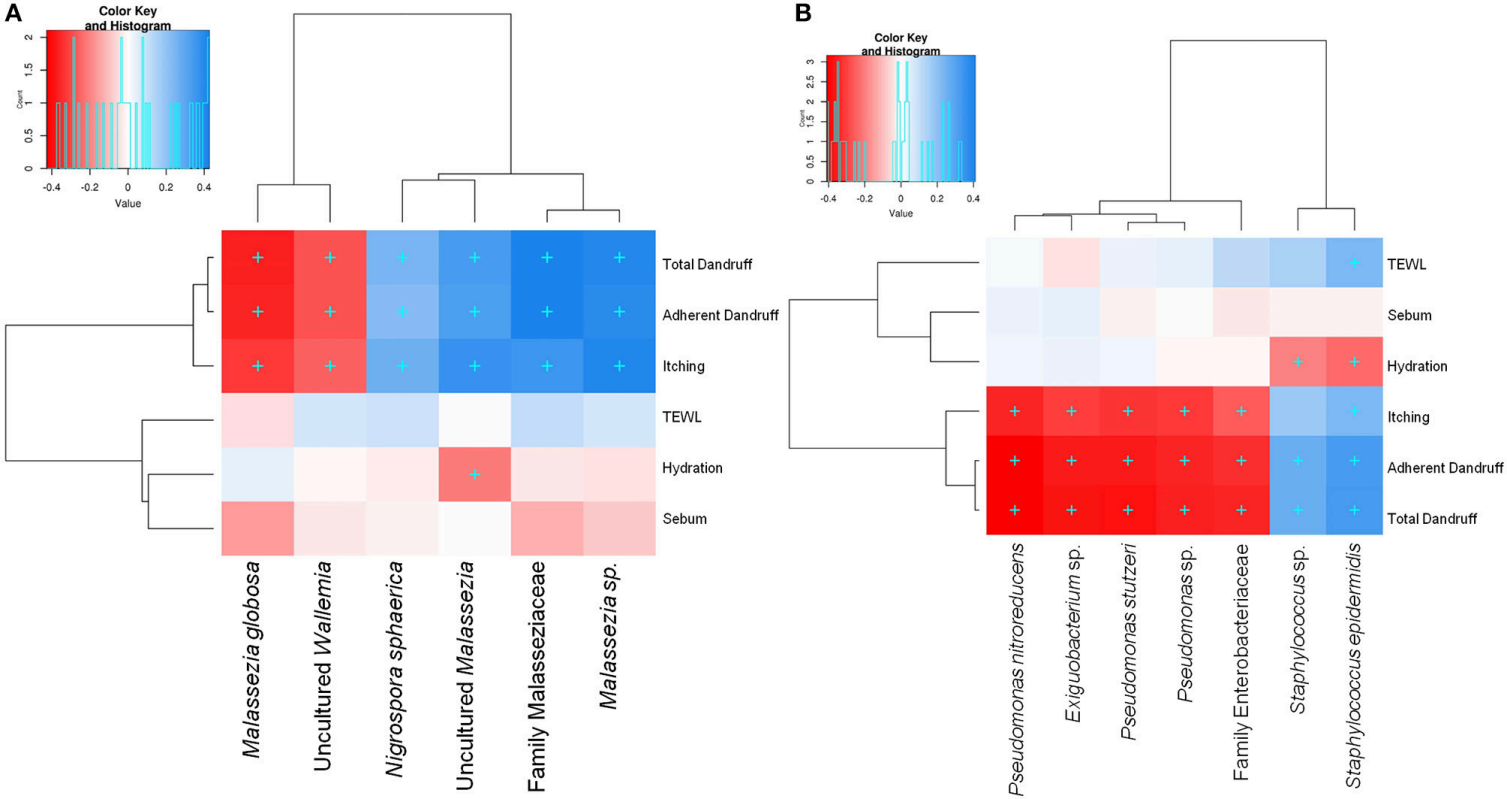
Spearman's correlation between microbial taxa and host physiological parameters. **(A)** Fungal and **(B)** bacterial taxa showing significant correlations (+, FDR adjusted *p* ≤ 0.05) with any of the parameters are plotted as a heatmap.

Among the three *Malassezia* subgroups, *Malassezia* sp. and uncultured *Malassezia* showed significant positive correlation (FDR adj. *p* ≤ 0.05) with dandruff scores and itching, whereas, *M. globosa*, which showed a higher abundance in the healthy scalp, was negatively correlated with these parameters (Figure 3A). Uncultured *Malassezia* also showed a significant negative correlation with hydration. These findings yet again suggest that the uncharacterized *Malassezia* subgroups share a positive relationship with dandruff.

Among the bacterial population, *S. epidermidis* and *Staphylococcus* sp. displayed a significant positive correlation (FDR adj. *p* ≤ 0.05) with dandruff scores, TEWL and itching (Figure 3B). These species also correlated negatively with hydration. Although being low in abundance, *Pseudomonas* spp. showed a negative correlation with these parameters. Other low abundant bacterial and fungal taxa also showed significant correlations with selected parameters.

### Functional analysis of the scalp microbiome

**Figure 4:**
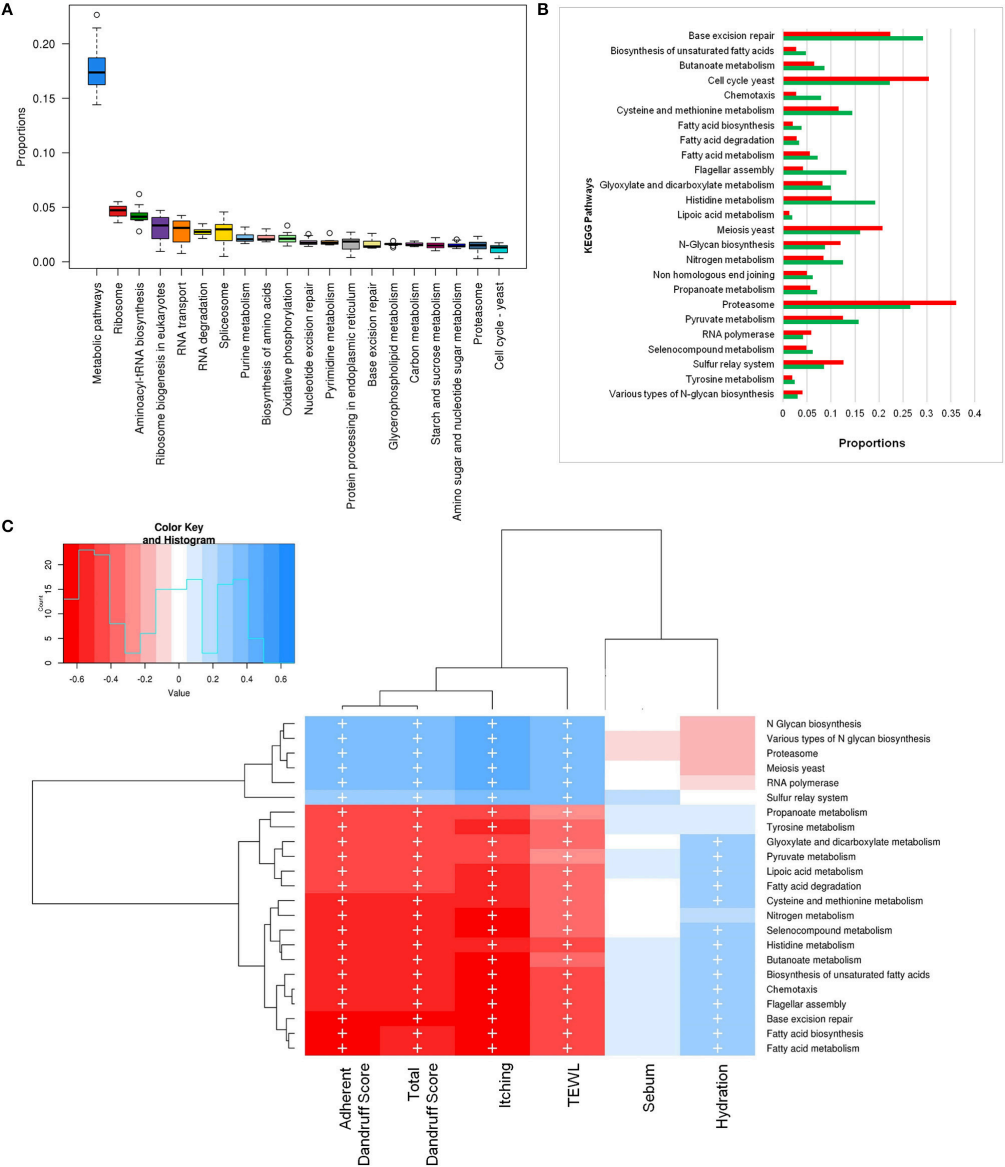
Functional analysis of fungal microbiome. **(A)** Twenty most abundant fungal metabolic pathways in the healthy scalp. **(B)** Differentially abundant (*p* ≤ 0.05) fungal pathways between healthy (green bars) and dandruff scalp (red bars). **(C)** Spearman's correlation between fungal metabolic pathways and host physiological parameters. Fungal taxa showing significant correlations (+, FDR adjusted *p* ≤ 0.05) with any of the parameters are plotted as a heatmap.

To investigate the functional potential of the scalp microbial communities, a comprehensive metagenomic analysis was also performed. A higher fungal metabolic (KO) diversity was observed in dandruff scalp compared to the healthy scalp (Figure S7), which was consistent with the higher alpha diversity of the fungal population in the dandruff scalp (Figure 1A). Several fungal KEGG pathways were observed to vary significantly (*p* ≤ 0.05, Wilcoxon test) between the healthy and dandruff scalp (Figures 4A,B). Amino acid metabolism pathways (histidine, cysteine, and methionine metabolism) and lipoic acid metabolism pathway were more abundant in healthy scalp than in dandruff scalp. Further, pathways for N-glycan biosynthesis, which are implicated in cell-host interaction, were enriched in the dandruff scalp (Dranginis et al., 2007).

Annotation of KEGG modules showed that the modules for N-glycan precursor biosynthesis and N-glycosylation were enriched in the dandruff scalp (Figure S8) and *M. restricta* was observed to significantly contribute to the N-glycan biosynthesis pathway (Figure S9). An interesting observation was the significant enrichment of pathways related to general functions and genetic information and processing (sulfur relay system, proteasome pathway, cell cycle in yeast, and meiosis) in dandruff scalp compared to healthy scalp. Correlation analysis suggested *M. restricta* to significantly contribute to these general function pathways (Figure S9). Other cellular eukaryotic pathways for genetic information processing (base excision repair, homologous recombination, mismatch repair, oxidative phosphorylation pathway, etc.) were enriched in the healthy scalp. The flagellar assembly and chemotaxis pathways were enriched in the healthy scalp and were majorly contributed by *M. obtusa* and *M. furfur*. A significant positive correlation of the essential cellular pathways (N-glycan biosynthesis, cell cycle in yeast, RNA polymerase, proteasome, RNA transport, etc.) was observed with dandruff-associated clinical parameters (dandruff score, itching and TEWL). In contrast, the other pathways such as amino acid and lipoic acid metabolism, displayed a negative correlation with these parameters (Figure 4C).

**Figure 5:**
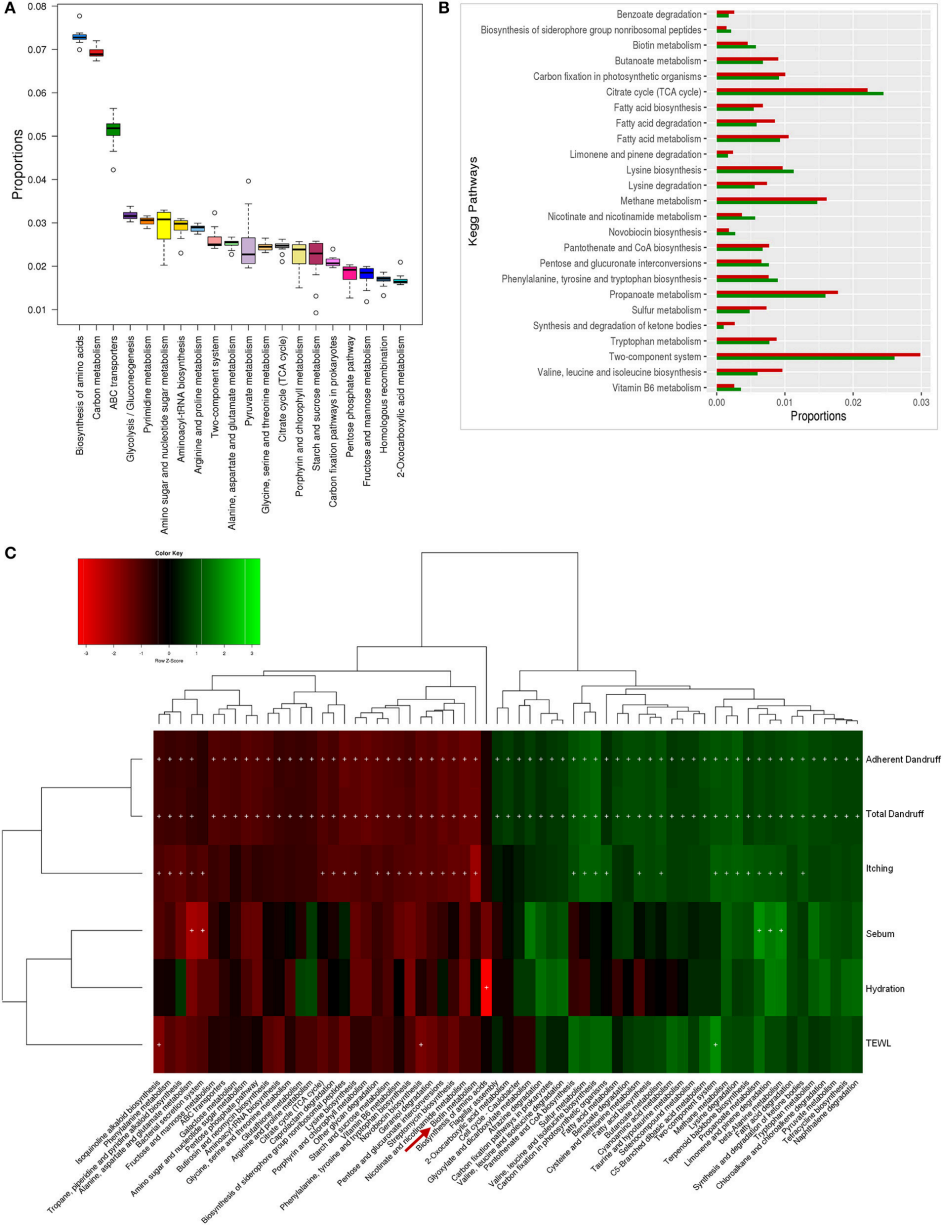
Functional analysis of bacterial microbiome. **(A)** Twenty most abundant bacterial metabolic pathways in the healthy scalp. **(B)** Differentially abundant (*p* ≤ 0.005) bacterial pathways between healthy (green bars) and dandruff scalp (red bars). **(C)** Spearman's correlation between bacterial metabolic pathways and host physiological parameters. Bacterial taxa showing significant correlations (+, FDR adjusted *p* ≤ 0.05) with any of the parameters are plotted as a heatmap.

The bacterial microbiome presented a higher number of KEGG metabolic pathways than the fungal microbiome, which showed significant variations (*p* ≤ 0.05, Wilcoxon test) between the healthy and dandruff scalp (Figures 5A,B) (Figure S10). Pathways related to vitamins and cofactors (biotin, porphyrin and chlorophyll, vitamin-B6, nicotinate and nicotinamide metabolism, ubiquinone and other terpenoid-quinone biosynthesis), and amino acids (alanine, aspartate, arginine, glutamate and proline and lysine metabolism and biosynthesis) were significantly higher in healthy scalp than dandruff scalp (Figure S11) and their KEGG modules also showed similar trends (Figure S12). These pathways correlated negatively with dandruff scores and itching (Figure 5C). A high abundance of antibiotic resistance and biosynthesis pathways was also observed in the microbiome (beta-lactam resistance, caprolactam degradation, streptomycin biosynthesis, novobiocin biosynthesis, tetracycline biosynthesis, and butirosin and neomycin biosynthesis), which showed significant variations between the healthy and dandruff scalp, and showed significant negative correlation with dandruff score and itching. The presence of these pathways was also reported in healthy skin sites in a previous study (Oh et al., 2014).

### Enrichment of biotin and vitamin metabolism pathways in the bacterial microbiome of the healthy scalp

The biotin metabolism pathway was observed to be significantly enriched (*p* = 0.0001, Wilcoxon test; Figure 5B) in the healthy scalp compared to the dandruff scalp. The three modules for biotin metabolism, M00123, M00573 (BioI), and M00577 (BioW), which are involved in the biosynthesis of biotin from Pimeloyl ACP/CoA were also enriched in the healthy scalp (Figure S12). Biotin is a necessary cofactor for carboxylase enzymes that play a major role in metabolic processes including glycolysis, fatty acid synthesis, and amino acid metabolism, and is an essential scalp nutrient for hair growth and scalp health (Patel et al., 2017). It is also reported to play a vital role in protein synthesis and more precisely in keratin production, which is essential for healthy hair growth (Chiu et al., 2015).

Though, the genes for complete biotin metabolism pathway were observed in the dataset, a detailed analysis showed an enrichment of the major KOs of biotin biosynthesis and its transporters in the healthy scalp compared to the dandruff scalp (Figures S13, S14). Interestingly, *Propionibacterium* spp., which was the most abundant bacterial species in the healthy scalp microbiome, also carries the genes for biotin biosynthesis and its transport, suggesting its key role in providing the host with biotin. A significant positive correlation between *Propionibacterium* spp. and the amino acids and vitamin metabolism pathways, including the biotin metabolism pathway was also observed (Figure S15). Biotin and other B-vitamins, such as vitamin-B6, nicotinate, and pantothenic acid are essential precursors for various enzymes that are required for vital biochemical reactions in the living cells (Leblanc et al., 2013). Humans and other mammals cannot synthesize biotin and thus, obtain the vitamin through exogenous sources (Said, 2008). The human host keratinocytes can transport (import) vitamins such as biotin, lipoic acid, and pantothenic acid, which are essential for a healthy skin or scalp surface, through the Na-dependent multivitamin transporters (SMVT), and also express a biotin-specific transport component (Grafe et al., 2003; Uchida et al., 2015). Thus, it can be hypothesized that the uptake of biotin and other vitamins synthesized by the scalp microbiome could occur by the scalp keratinocytes for scalp nutrition, which can contribute in maintaining a healthy scalp state.

## Discussion

This study provides the first comprehensive overview of the scalp microbiome and its association with healthy and dandruff scalp using complementary high throughput sequencing approaches (bacterial 16S rRNA and fungal ITS1 amplicon sequencing, and shotgun metagenomic sequencing) in a large human cohort. *Propionibacterium acnes* and *Staphylococcus epidermidis* emerged as the major bacterial colonizers in the Indian population, similar to the previous reports from other world populations, where the former was found associated with healthy scalp and the latter with dandruff scalp (Clavaud et al., 2013; Wang et al., 2015; Soares et al., 2016; Xu et al., 2016). The ratio of *P. acnes* to *S. epidermidis* was higher in healthy scalp compared to the dandruff scalp, which corroborates with previous scalp microbiome studies carried out on French, Chinese, and Brazilian population (Clavaud et al., 2013; Wang et al., 2015; Xu et al., 2016). These results and the results from the taxonomic analysis support the antagonistic relationship between *S. epidermidis* and *P. acnes*, as observed in previous studies (Xu et al., 2016). Therefore, it can be inferred that the ratio of *P.acnes* to *S. epidermidis* might be a crucial biomarker for the diagnosis and prevention of the dandruff condition. *M. restricta* and to a lower extent *M. globosa* were the major fungal colonizers on the scalp, and a lower *M. restricta* to *M. globosa* ratio was found to be associated with the healthy scalp. Similar ratio was also reported on a Chinese cohort using a similar sequencing (Illumina MiSeq) technology (Xu et al., 2016). However, a few other scalp microbiome studies have shown contrasting results, which could be attributable to the lower size of the cohort, differences in population origins and usage of traditional sequencing technologies (Gemmer et al., 2002; Park et al., 2012; Clavaud et al., 2013; Soares et al., 2016).

One of the significant results from the study was the high abundance of uncultured *Malassezia* sp. in dandruff scalp microbiome (25.26%) compared to the healthy scalp (14.44%), and its significant positive correlation with dandruff-associated parameters. A similar, but lower (< 4% of the total reads) presence of OTUs corresponding to uncultured *Malassezia* sp. was also noted in a recent study carried out in a Chinese cohort and Brazilian cohort (Soares et al., 2016; Xu et al., 2016). A higher abundance and strain-level variations of uncharacterized *Malassezia* have also been observed previously in dandruff, seborrheic dermatitis, and psoriasis (Tajima et al., 2008; Hiruma et al., 2014; Xu et al., 2016; Tett et al., 2017). It is interesting to note that different species of *Malassezia* differ in their pathogenicity and adapt to various sites on the human skin, which is primarily attributable to factors such as the host physiological conditions (sebum, immune response, sweat, etc.), environment (temperature, humidity, UV exposure), and presence of other microbial inhabitants (Wu et al., 2015; Grice and Dawson, 2017; Zhu et al., 2017). Due to the inherent diversity in ethnicity, climatic conditions, diet, and other factors, a distinct microbial diversity was anticipated in the Indian population, which was evident from the high abundance of uncultured *Malassezia* sp. in the scalp microbiome (Pouradier et al., 2017). These observations necessitate the strain-level *in vitro* characterization of the unknown *Malassezia* species to identify population-specific biomarkers, and to understand their possible role in the etiology of dandruff in the various populations. Although the species of the core microbiome showed high abundance in majority of the individuals, a high inter-individual variation was also noticed across all the samples. This variation is attributable to the differences in the host physiological conditions and other environmental factors, which is also reported in previous studies (Oh et al., 2014; Soares et al., 2016).

Here, we also carried out a systematic measurement of parameters associated with the host physiology. *M. globosa* showed a negative correlation with dandruff score and itching, which further supports its role in the healthy scalp in this study and another study from the Chinese population (Xu et al., 2016). Further, the dandruff-associated bacterial species, *S. epidermidis*, showed a significant positive correlation with dandruff score (adherent and total), TEWL and itching.

For the first time, the present study provides insights into the yet unknown functional potential of scalp microbiome in healthy and dandruff conditions. The N-glycan biosynthesis pathway, which is essential for maintaining the fungal cell wall integrity and for glycoprotein biosynthesis, was enriched in the dandruff scalp (Mora-Montes et al., 2007). These glycoproteins are used by the fungal cells for adherence to the host surface, and thus the enrichment of this pathway suggests its role in facilitating the fungal growth on the scalp (Mcdowell et al., 2013). This suggests that the adherence of the *Malassezia* species, which is linked to the impairment in the scalp barrier function of the stratum corneum may further help in the development of *Malassezia* in the dandruff areas of the scalp. However, even though innate immune receptors such as TLR2 have been reported to mediate *Malassezia* sp. cytotoxicity to keratinocytes *in vitro*, its detailed mechanism remains to be identified in *in vivo* (Donnarumma et al., 2014; Cavusoglu et al., 2016). A high abundance of pathways related to general functions and genetic information and processing (sulfur relay system, proteasome pathway, cell cycle in yeast, and meiosis) was also observed in the dandruff scalp compared to the healthy scalp, which showed a significant positive correlation with *M. restricta*. The “cell cycle yeast” and “meiosis” are vital pathways involved in the fungal cell cycle and proliferation, and their higher abundance on the dandruff scalp suggests their role in the enhanced proliferation of *Malassezia* sp. on the dandruff scalp. According to recent reports, the “proteasome pathway” is linked to capsule formation, which is a major virulence determinant in fungi (Geddes et al., 2016). Also, the “sulfur system” has been linked to fungal virulence through the mitochondrial Fe-S cluster protein, which is reported to influence the mitochondrial iron metabolism, cell wall resistance, and capsule formation in fungi (Do et al., 2015; Park et al., 2018). Previous reports on comparative genomics and functions of *M. restricta* are limited to certain extracellular digestion processes like lipases and cell wall biosynthesis, but the direct link between the virulence factors and these pathways (proteasome and sulfur systems) remains to be investigated in dandruff (Stalhberger et al., 2014; Sommer et al., 2015; Wu et al., 2015; Park et al., 2017). Nonetheless, the results from this study provide clues for the role of these pathways in the manifestation of dandruff condition on the scalp, and future confirmatory studies on *M. restricta* could be helpful in developing novel anti-fungal strategies to combat dandruff.

A significant enrichment of biosynthesis and metabolism pathways of vitamins and cofactors was observed in the bacterial microbiome of healthy scalp compared to the dandruff scalp. Many of these vitamins (biotin) and amino acids are reported to play a beneficial role in maintaining healthy hair and scalp and in controlling dandruff, (Nisenson, 1969; Rushton, 2002). Nicotinate and nicotinamide metabolism pathway is involved in the vitamin-B3 (niacin) production, which helps in the maintenance of scalp health and in increasing the hair diameter (Magnúsdóttir et al., 2015). Vitamin-B6 and B12 are essential supplements for improving hair structures and are used in the treatment of certain dermatological conditions, such as psoriasis and dermatitis (Dranginis et al., 2007; Biagi et al., 2016). Further, the lysine biosynthesis pathway showed similar enrichment, which is an essential amino acid known to restore scalp condition and reduce hair loss (Rushton, 2002). The negative correlation with dandruff-associated parameters pathways with physiological parameters suggested that the vitamin and amino acids (biotin, vitamin-B6, nicotinate, and lysine) show a negative correlation with dandruff-associated parameters. Taken together, these results highlight the possible beneficial role of bacterial scalp microbiome in supplying essential vitamins and amino acids to the host, which seems similar to the role played by the human gut bacteria in host health (Magnúsdóttir et al., 2015) (Figure S16).

This study provides the first insights into the functional potential of microbiome in healthy and dandruff scalp. The key findings anticipate the imperative role of the yet uncharacterized and uncultured *Malassezia* sp. in dandruff. An interesting addendum here is that while a few species from the fungal microbiome appear to strongly influence the incidence of dandruff, a few species from the bacterial microbiome are involved in providing the scalp with essential nutrients to maintain a healthy state. Thus, the results from this study expand our understanding of the dandruff scalp microbiome and provide new perspectives on the pathogenesis of dandruff.

## Author contributions

CC, NM, and VS conceived the idea. CC, NR, LB, NM, and VS designed the study. RS, CC, NM, and VS designed the experiments. PH, MV, and SS prepared the clinical protocol and carried out clinical sampling. RS carried out the experimental processing and sequencing of the DNA samples. LS performed statistical analysis of the clinical end-points. PM performed the computational analysis of amplicon data. PM and DBD performed the computational analysis of the metagenomic data. PM, DD, and RS carried out the statistical analysis of amplicon and metagenomic data and its correlation with the clinical parameters. RS, PM, CC, DD, NM, and VS interpreted the results and prepared the figures and tables. RS, PM, CC, DD, NR, LB, NM, and VS prepared the manuscript. All authors read and approved the final manuscript.

### Conflict of interest statement

CC, LS, LB and NM were employed by company L'Oréal Research & Innovation, France. PH, MV, SS and NR were employed by company L'Oréal India Pvt. Ltd., India. The remaining authors declare that the research was conducted in the absence of any commercial or financial relationships that could be construed as a potential conflict of interest.
