# Supplementary material for: Comparison of Healthy and Dandruff Scalp Microbiome Reveals the Role of Commensals in Scalp Health
Source: Front Cell Infect Microbiol. 2018 Oct 4;8:346. doi: 10.3389/fcimb.2018.00346 (PMC6180232; doi:10.3389/fcimb.2018.00346)
Supplement: Supplementary file 1 [file Data_Sheet_1.DOCX]

**Supplementary Information**

**Title:** Comparison of healthy and dandruff scalp microbiome reveals the role of commensals in scalp health

**Authors:** Rituja Saxena^1†^, Parul Mittal^1†^, Cecile Clavaud^2†^, Darshan B Dhakan^1^, Prashant Hegde^3^, Mahesh M Veeranagaiah^3^, Subarna Saha^3^, Luc Souverain^2^, Nita Roy^3^, Lionel Breton^2^, Namita Misra^2,3^*, Vineet K Sharma^1^*

**Affiliation:** ^1^Department of Biological Sciences, Indian Institute of Science Education and Research Bhopal, India, ^2^L’Oréal Research & Innovation, France and ^3^L’Oréal India Pvt. Ltd., India

*Corresponding authors

Vineet K Sharma: [vineetks@iiserb.ac.in](mailto:vineetks@iiserb.ac.in)

Namita Misra: [NMISRA@rd.loreal.com](mailto:NMISRA@rd.loreal.com)

†These authors contributed equally to this work

***Supplementary Figures***


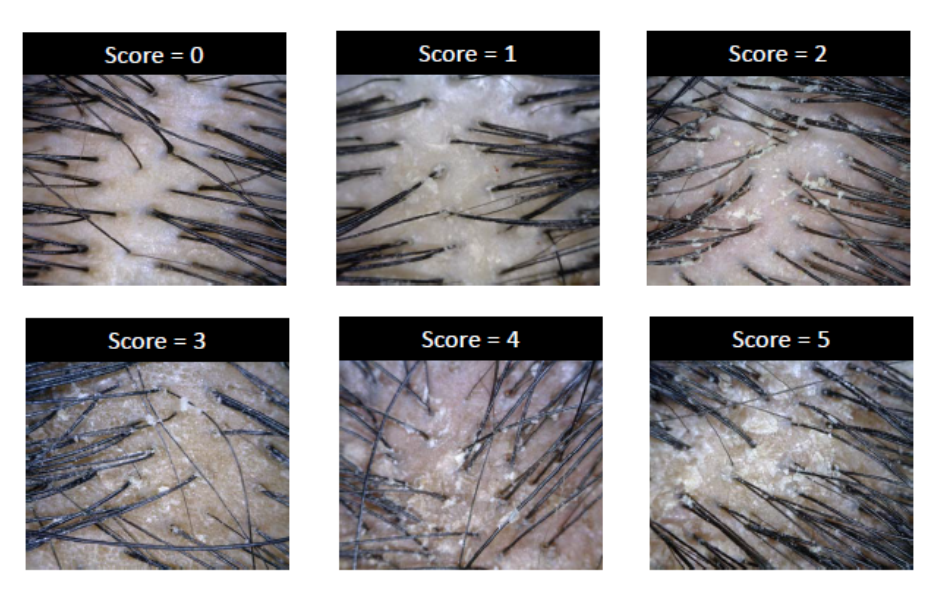


**Figure S1. Dandruff scoring as per the modified Van Abbe’s method.** Score 0 = no dandruff, 1 = minimal level of dandruff to 5 = highest level of dandruff


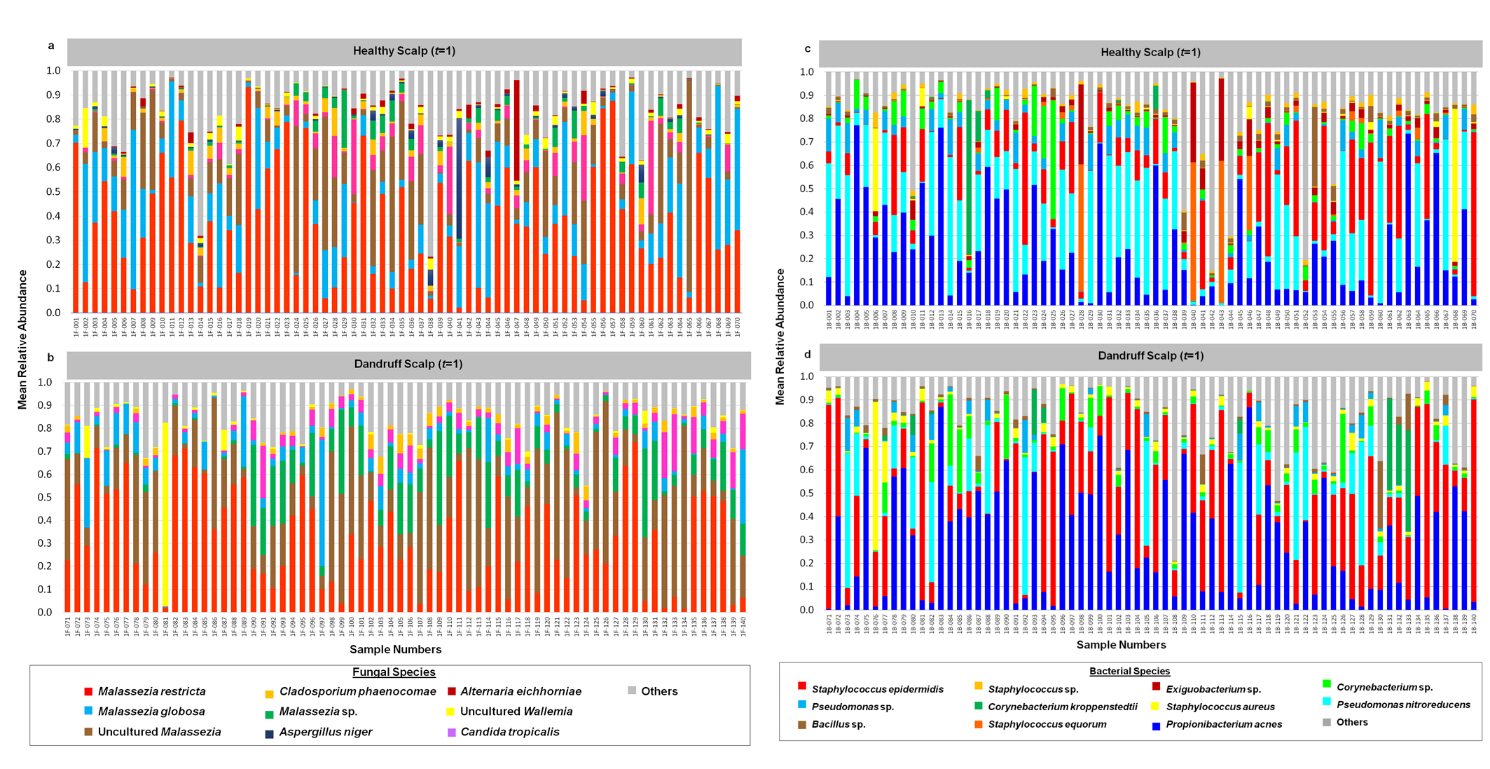


**Figure S2. Composition of (a and b) fungal and (c and d) bacterial species in healthy and dandruff scalp.**


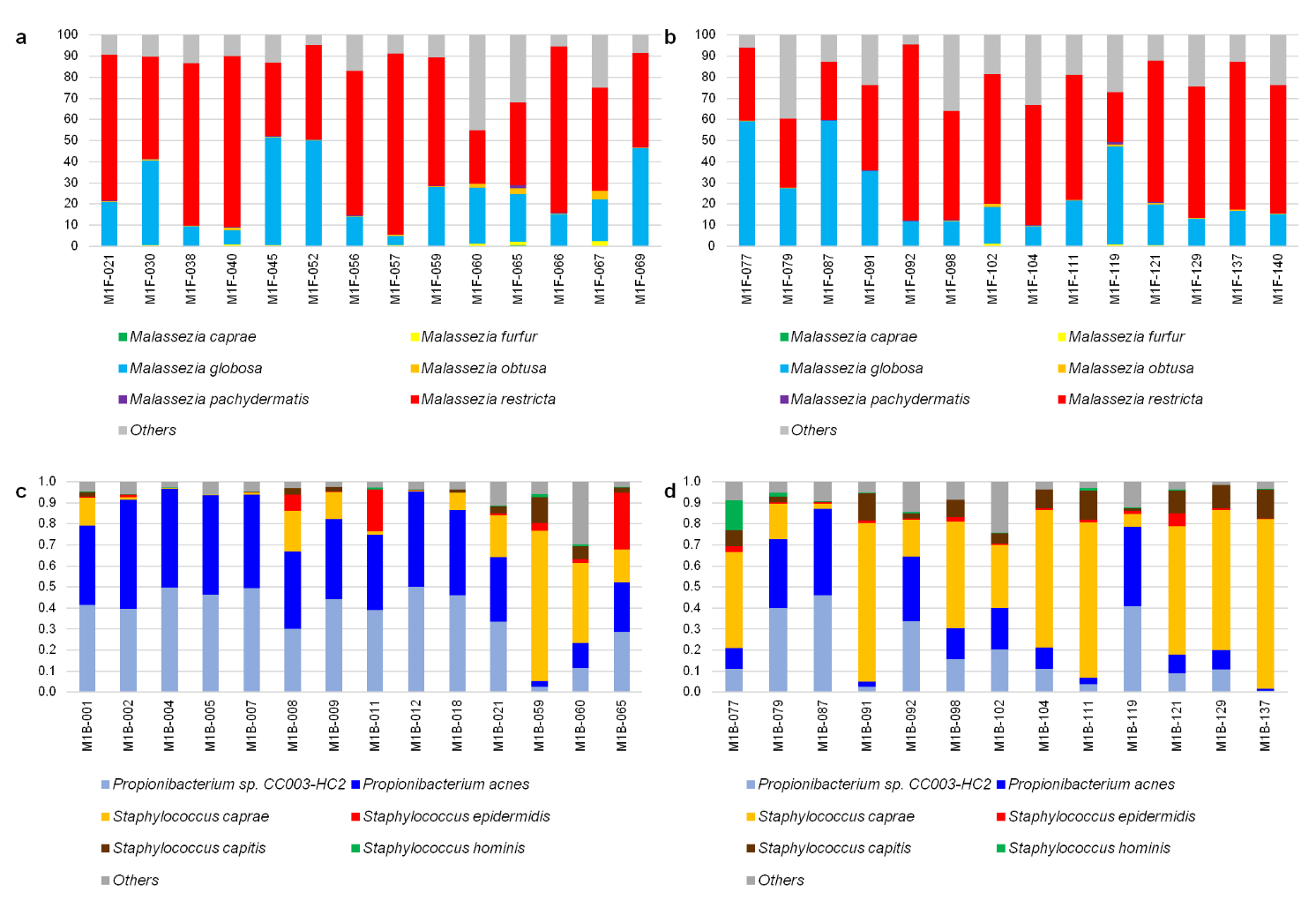


**Figure S3. Microbial community observed at the baseline using metagenomic data.** Distribution of *Malassezia* spp. in (a) healthy and (b) dandruff groups. Distribution of bacterial species in (c) healthy and (d) dandruff groups (species with mean abundance >1% are considered).


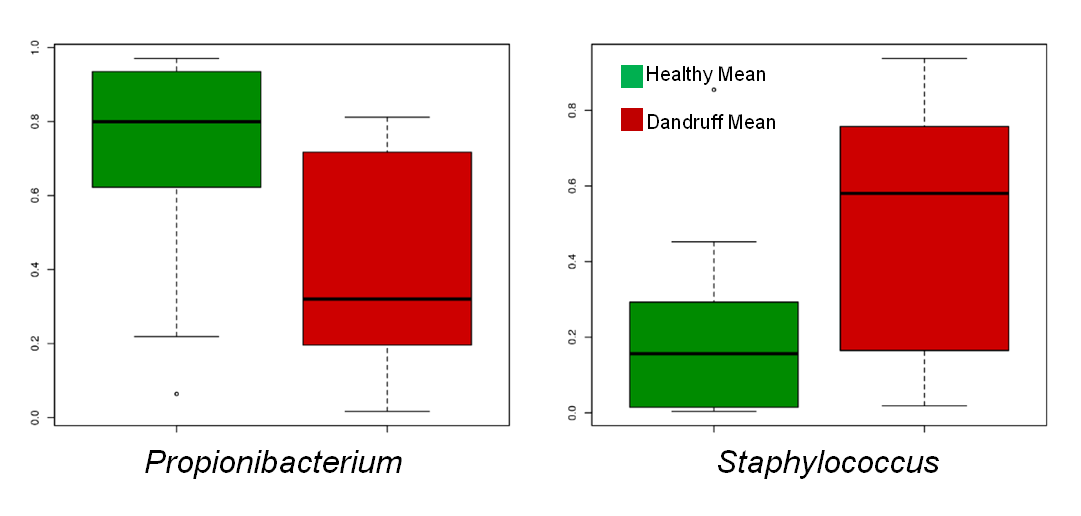


**Figure S4. Significant variation (*p*≤0.05) in the abundance of *Propionibacterium* and *Staphylococcus* spp. in healthy and dandruff scalp at the baseline.**


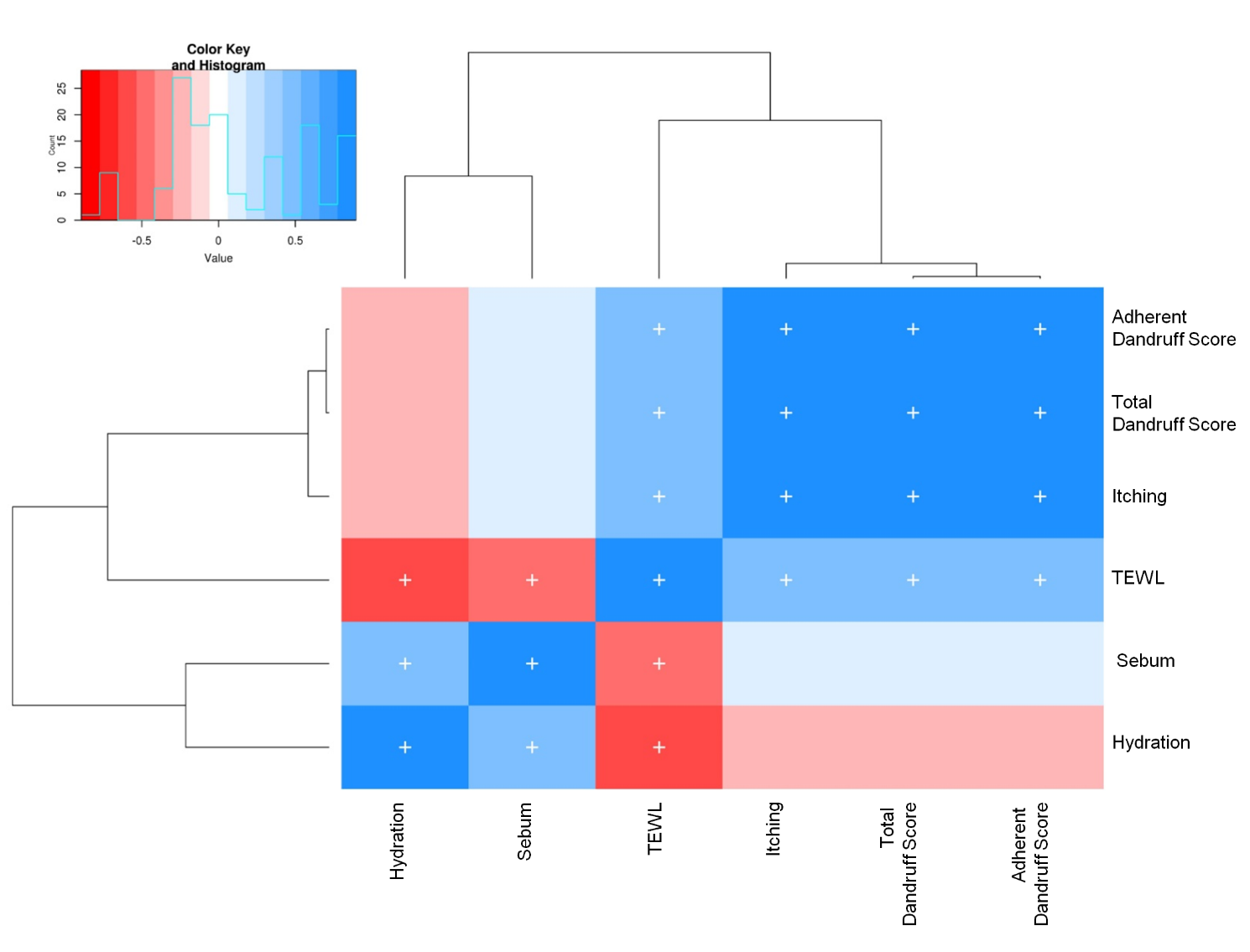
**Figure S5. Spearmann’s correlation between the host clinical parameters (+, *p*≤0.05).**


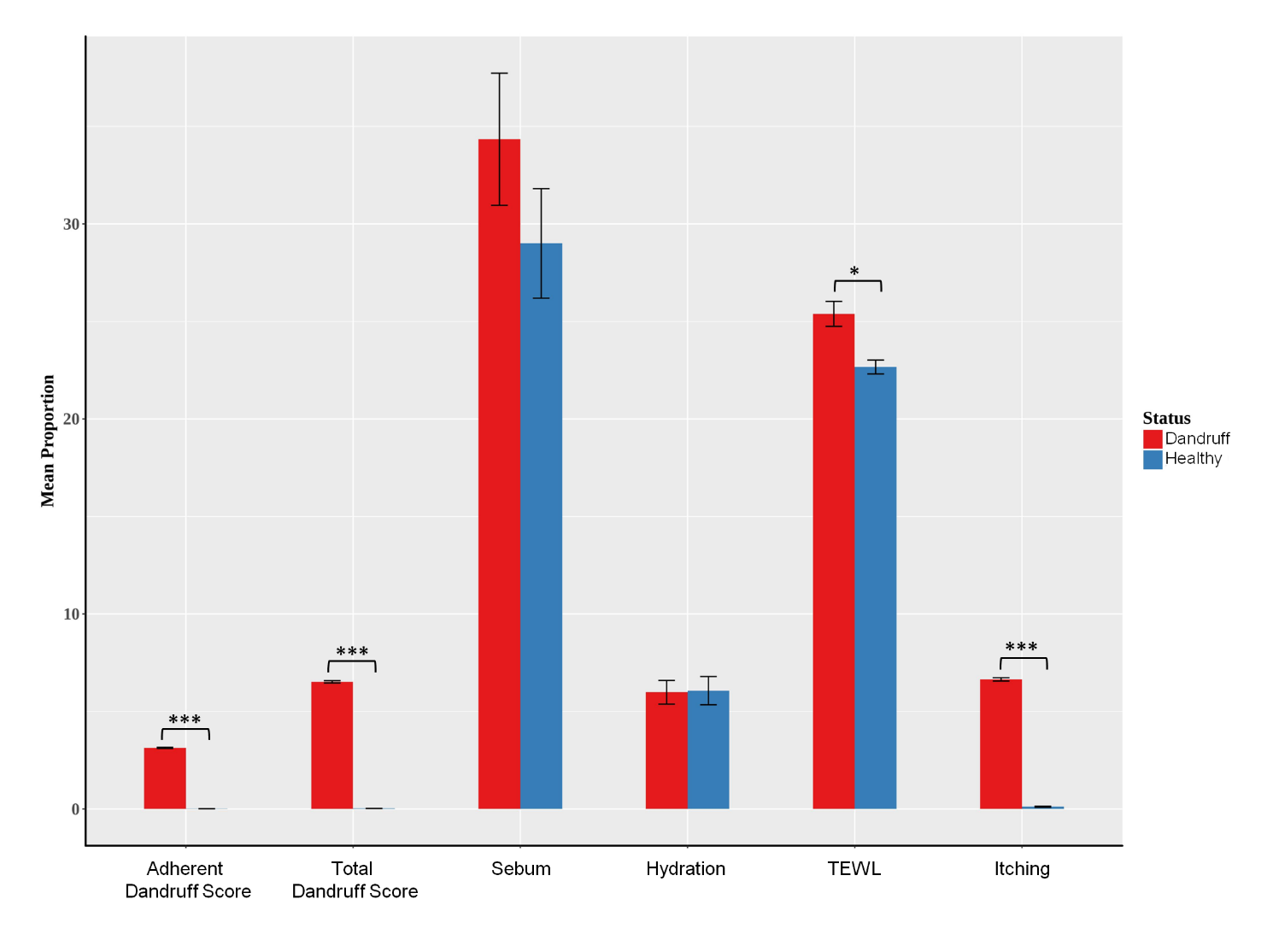


**Figure S6. Comparison of host clinical parameters between the healthy and dandruff groups (*: *p*≤0.05, ***: *p*≤0.0001).**


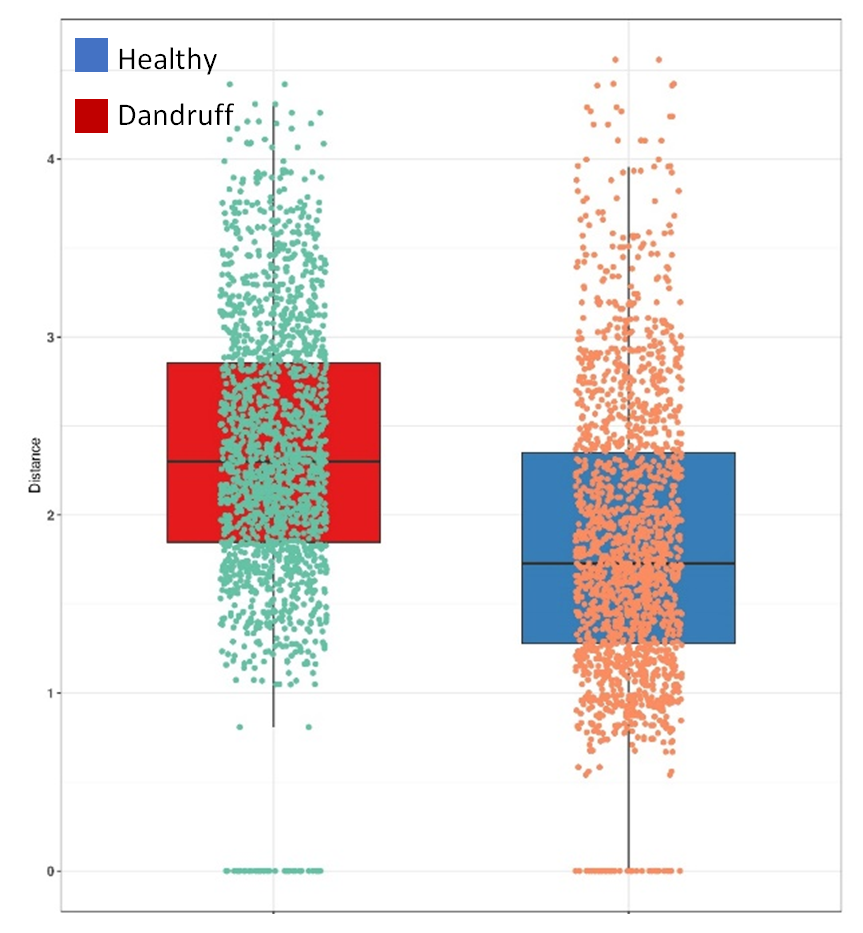


**Figure S7. Inter-sample distance plot (based on KOs of fungal microbiome) between healthy and dandruff scalp at the baseline.**


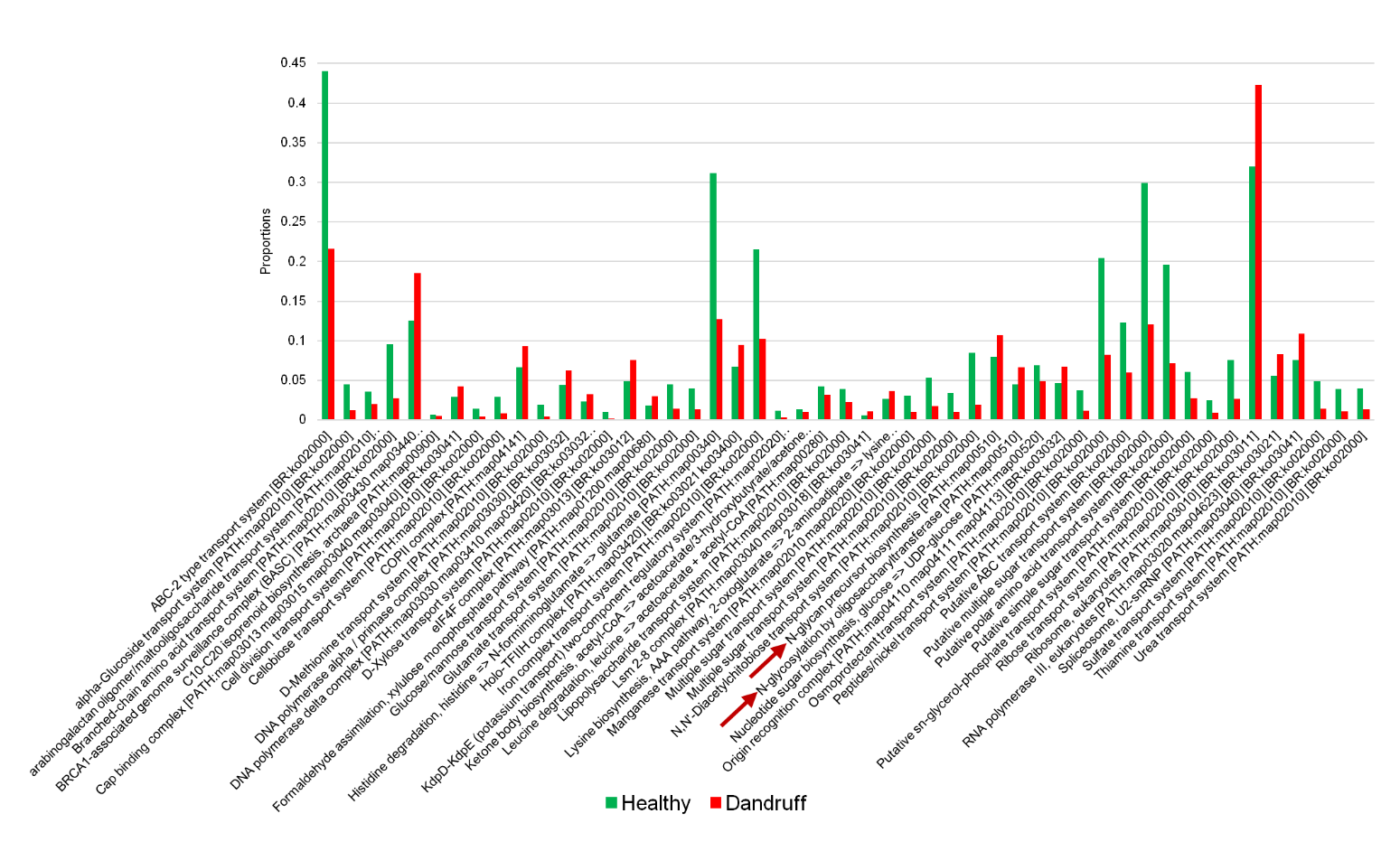


**Figure S8. Differentially abundant KEGG modules (fungal) between healthy and dandruff group at baseline in the fungal microbiome (*p*≤0.05).**


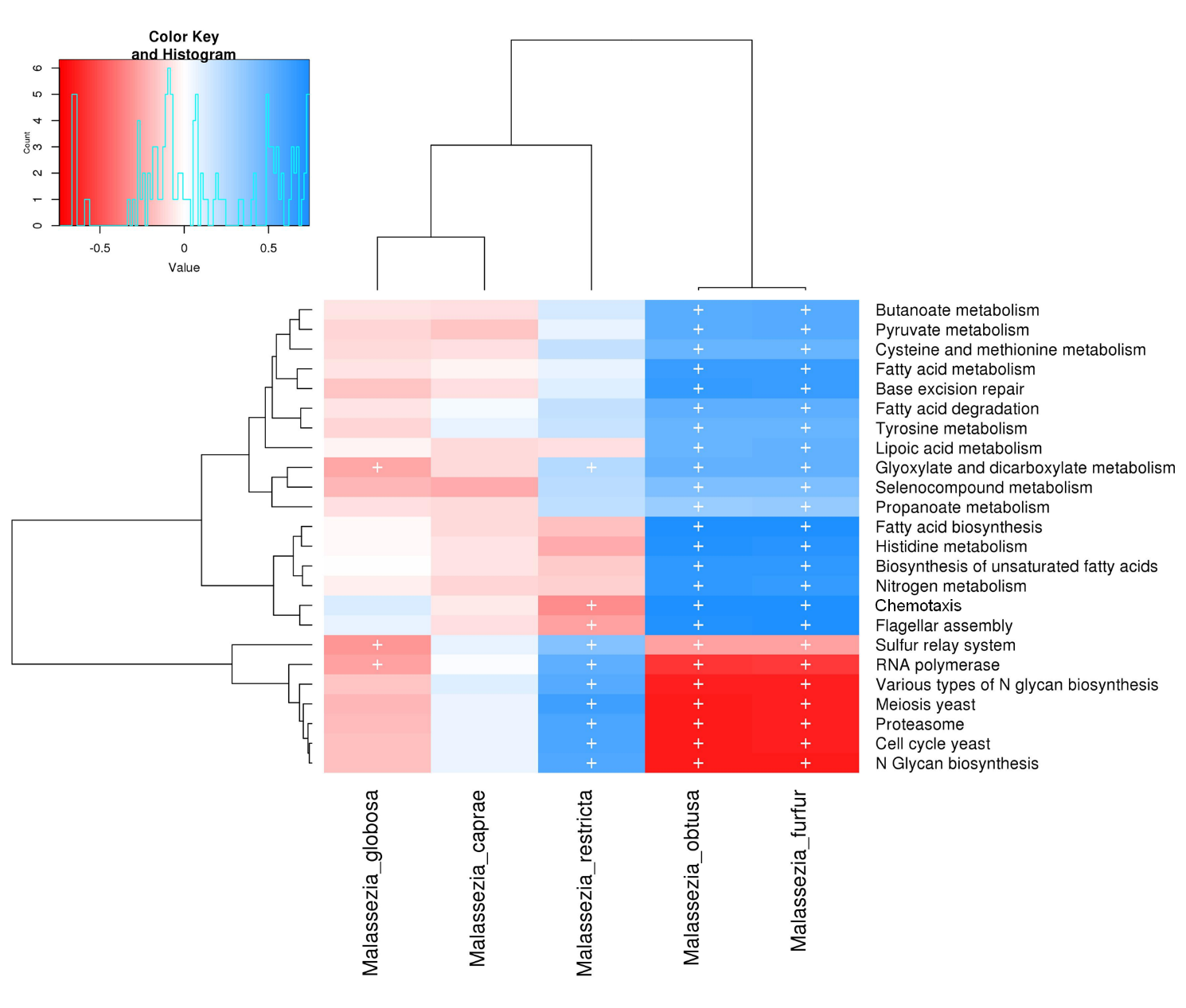


**Figure S9. Spearman’s correlation analysis of fungal pathway abundance with the abundance of *Malassezia* spp. to infer its taxonomic origin (+, *p*≤0.05).**


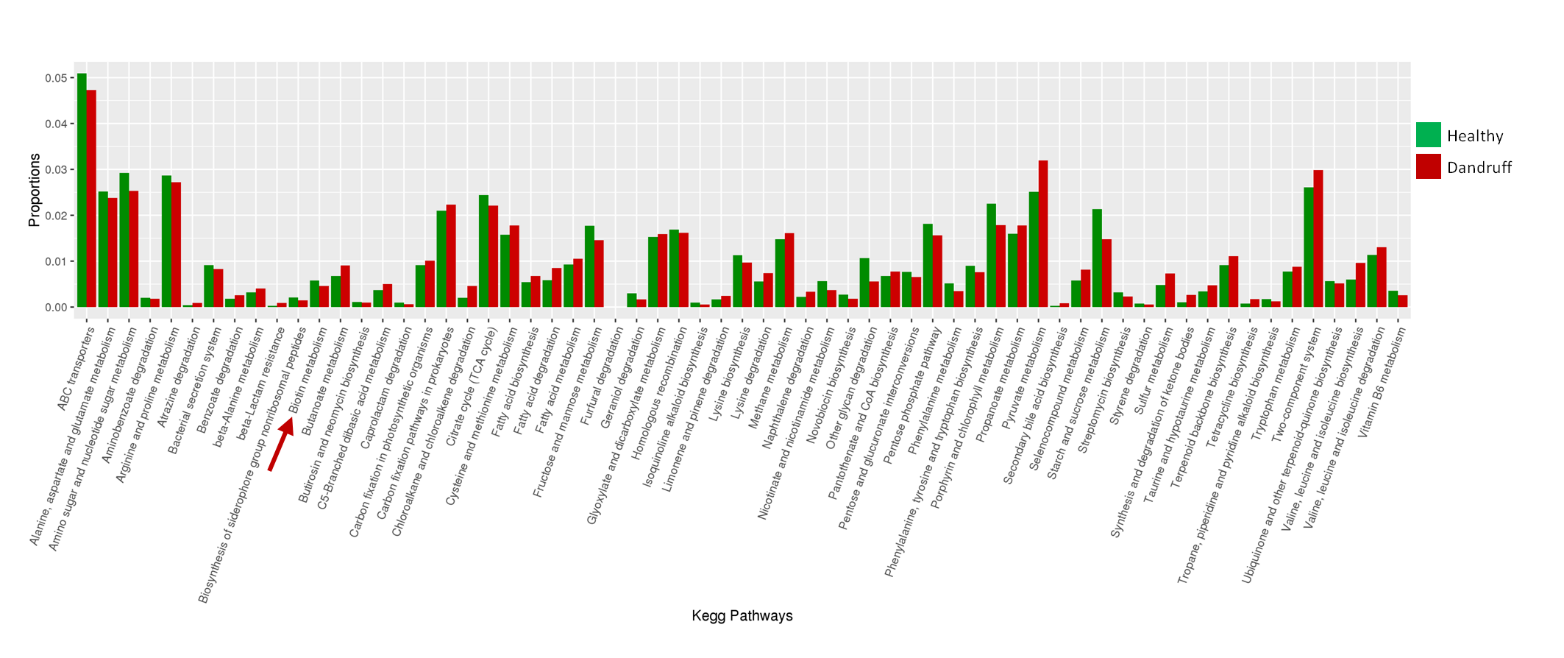


**Figure S10. Differentially abundant bacterial KEGG pathways in healthy and dandruff scalp at the baseline (*p*≤0.05).**


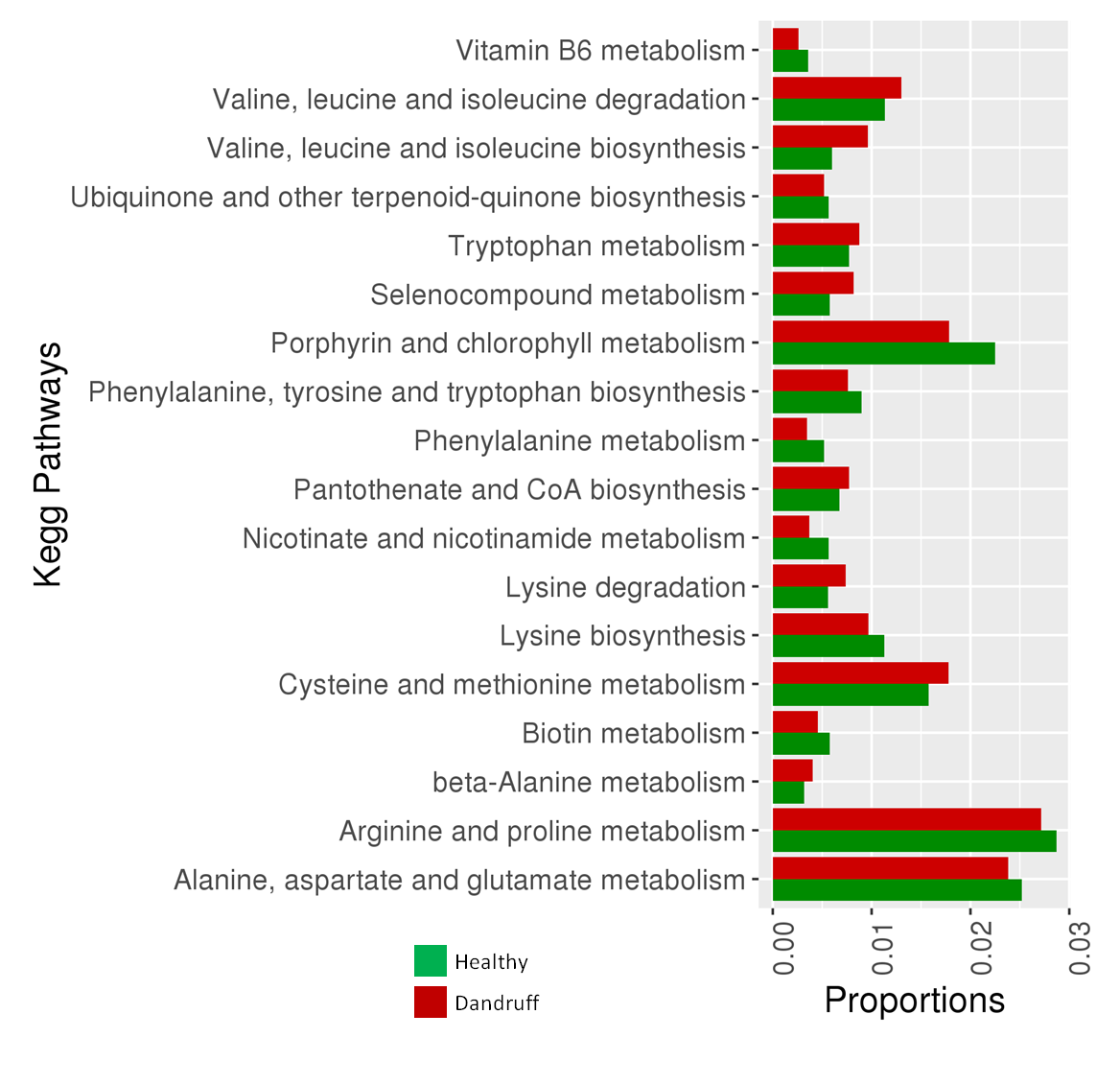


**Figure S11. Differentially abundant bacterial KEGG pathways related to amino acids, vitamins and cofactors in healthy and dandruff scalp at the baseline (*p*≤0.05).**


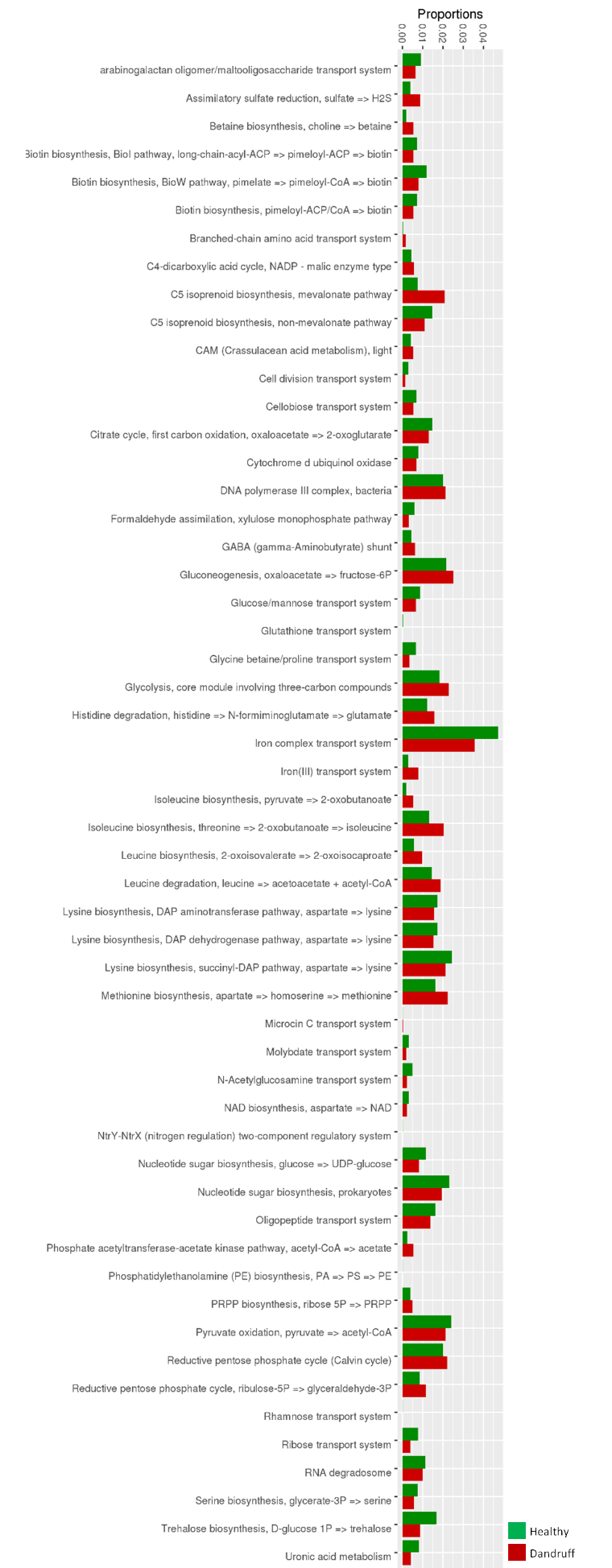


**Figure S12. Differentially abundant KEGG modules (bacterial) between healthy and dandruff scalp at the baseline (*p*≤0.05).**


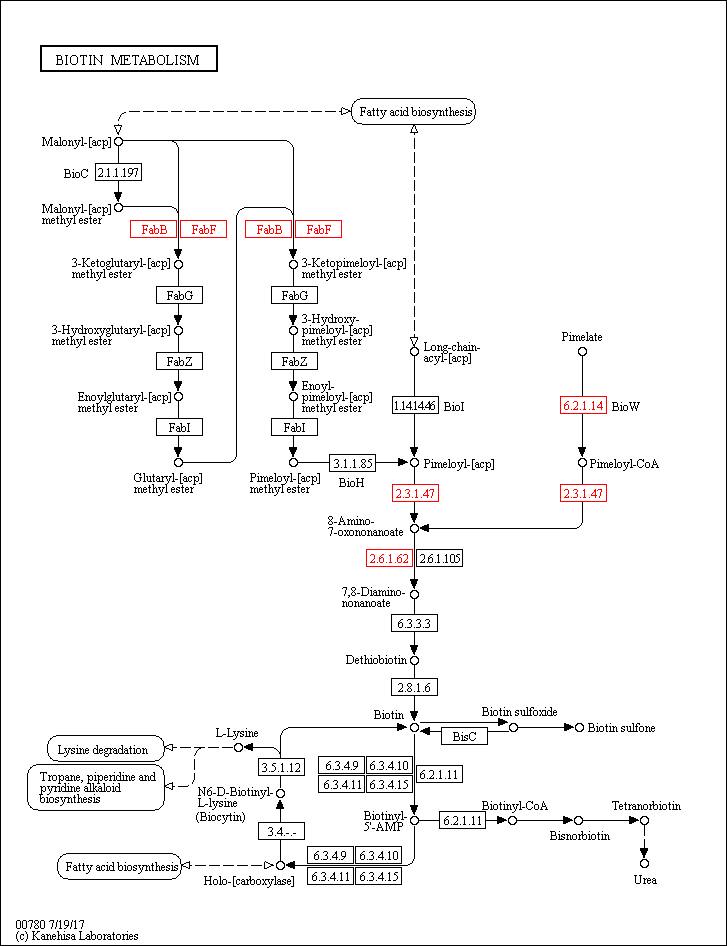
**Figure S13. KEGG pathway of biotin metabolism observed in the bacterial microbiome.** The KOs highlighted in red are enriched in the healthy scalp compared to the dandruff scalp.


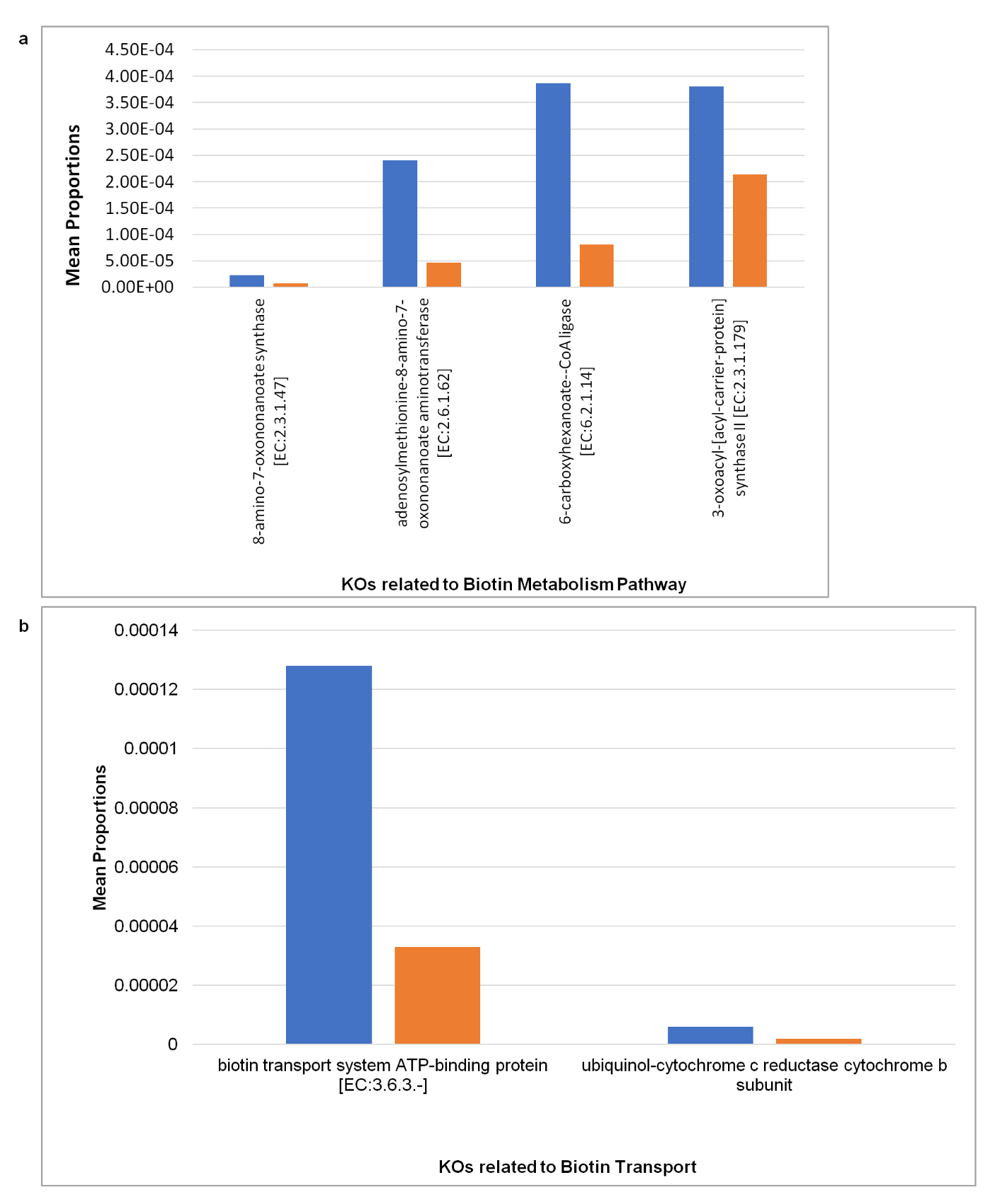
**Figure S14. (a) Significant variations in the KOs related to biotin metabolism observed between healthy and dandruff scalp (*p*≤0.05, Wilcoxon test). (b) Significant variations in the KOs related to biotin transport observed between healthy and dandruff scalp (*p*≤0.05, Wilcoxon test).**


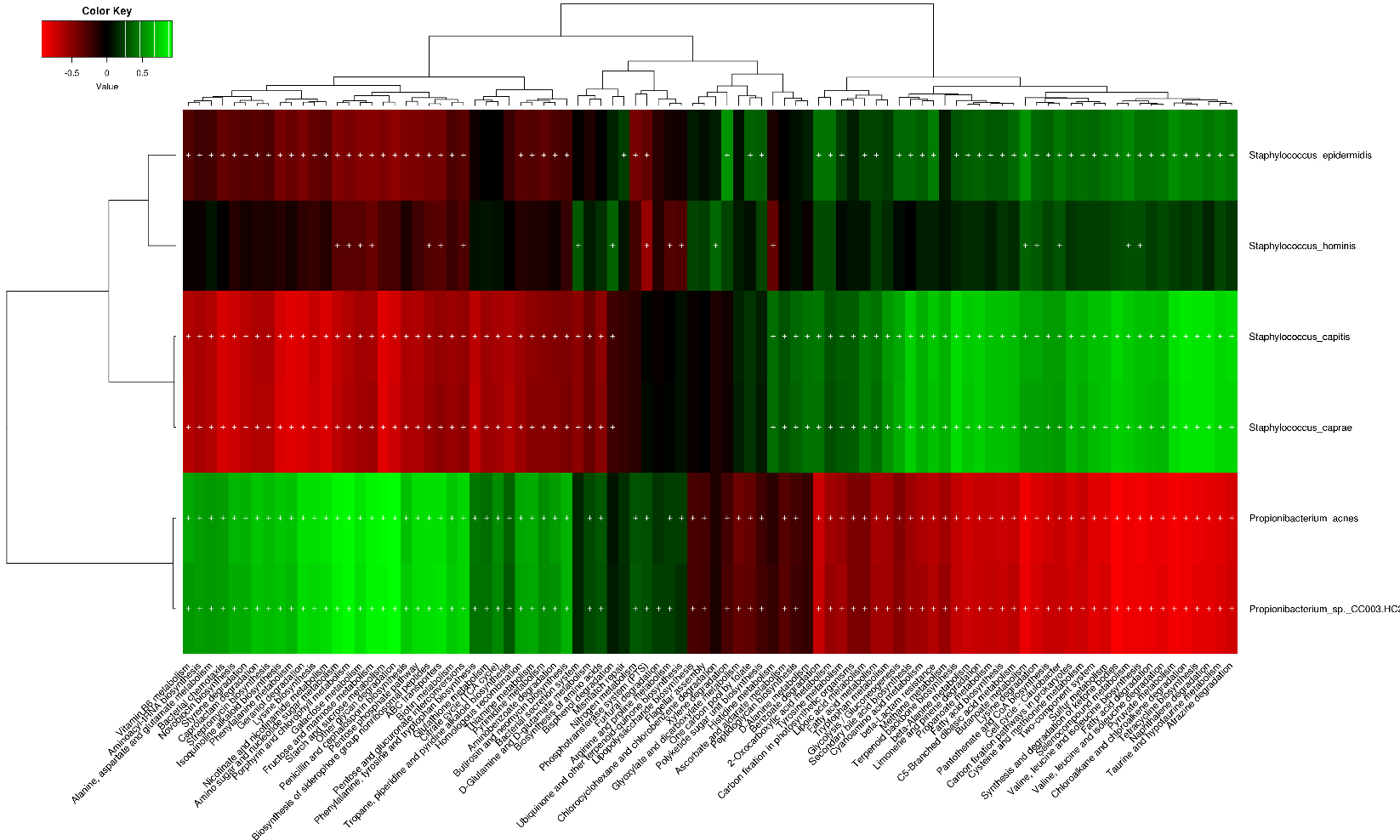
 **Figure S15. Spaermann’s correlation analysis of bacterial pathway abundance with species abundance to infer its taxonomic origin (+: *p*≤0.05).**


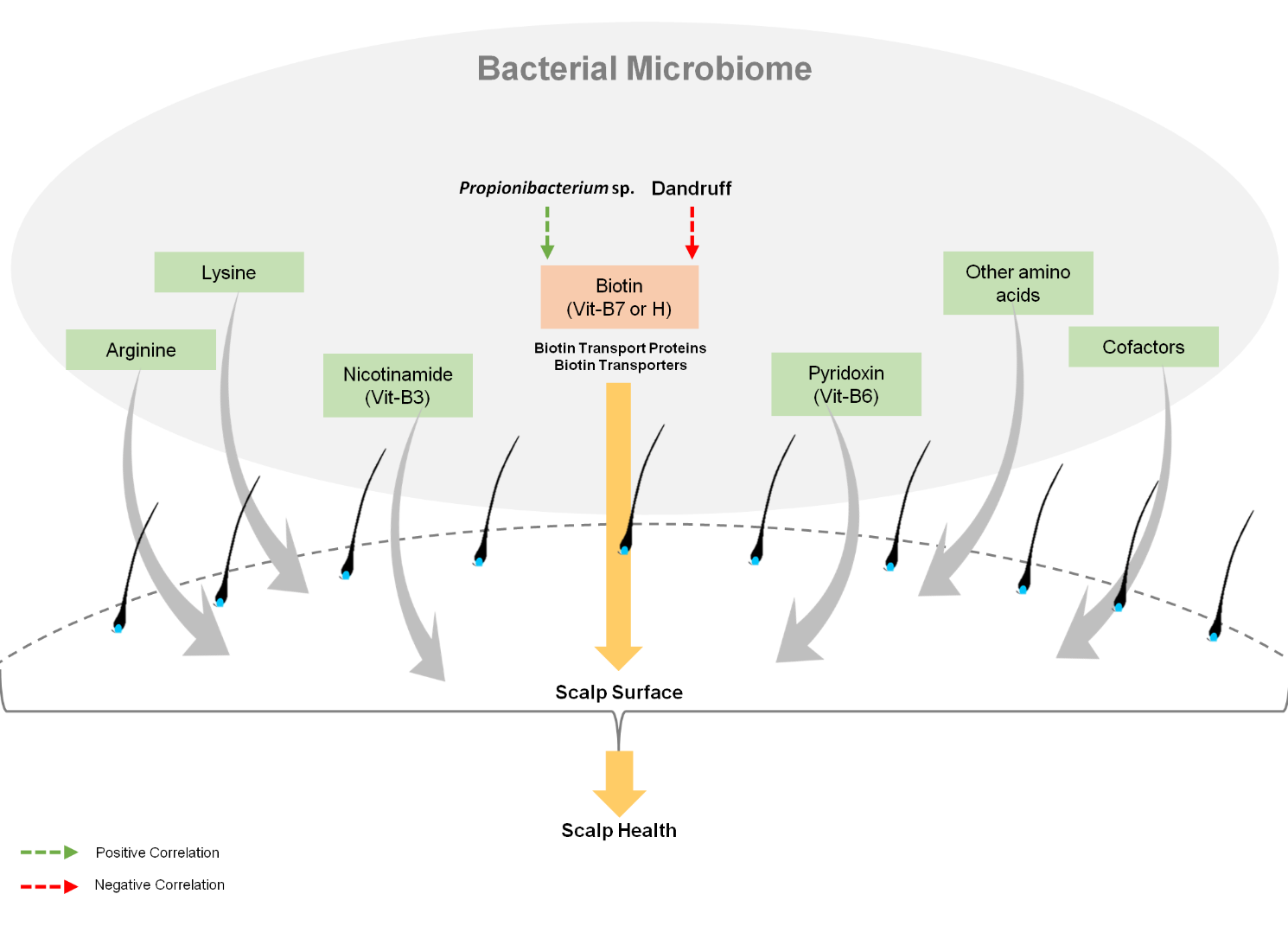
**Figure S16. Illustration of the mechanistic interplay observed between the scalp surface and the bacterial microbiome.** The vitamins and amino acids synthesised by the microbiome were observed as a useful source of nutrients for the scalp. Among these, biotin was observed to be majorly contributed by Propionibacterium spp. and the abundance of biotin metabolism pathway significantly increased after oil-treatment and reduced with the increase in dandruff.

***Supplementary Methods***

**PCR amplification of bacterial 16S rRNA V3 region and fungal ITS1 region**

The amplification of bacterial 16S rRNA V3 region was performed using Illumina Nextera XT adapter-ligated eubacterial V3 region-specific primers, 341F and 534R, with five different base modifications ([Wang and Qian, 2009](#_ENREF_9);[Soergel et al., 2012](#_ENREF_6)). Nucleotide bases were introduced in different numbers to increase the overall sequence diversity of the samples, thus improving the quality of the sequenced data. Bacterial DNA samples were divided into six groups and amplified using the six different primers. However, since ITS1 sequences are quite diverse across the fungal species ([Wang et al., 2015](#_ENREF_8)), this approach was not used for fungal ITS1 amplification.

Primer sequences for amplification of bacterial 16S rRNA V3 region are as below (the base inclusions are marked in bold):

The underlined regions in all the primer sequences are the Illumina Nextera XT adapter overhangs, whereas the non-underlined regions are the primer sequences known to target eubacterial 16S rRNA V3 region or fungal ITS1 region, respectively.

1. 341F-ADA

5’ TCGTCGGCAGCGTCAGATGTGTATAAGAGACAGCCTACGGGAGGCAGCAG 3’

534R-ADA

5’ GTCTCGTGGGCTCGGAGATGTGTATAAGAGACAGATTACCGCGGCTGCTGGC 3’

1. 341F_ADA_1B

5’ TCGTCGGCAGCGTCAGATGTGTATAAGAGACAG**T**CCTACGGGAGGCAGCAG 3’

534R_ADA_1B

5’ GTCTCGTGGGCTCGGAGATGTGTATAAGAGACAG**C**ATTACCGCGGCTGCTGGC 3’

1. 341F_ADA_2B

5’ TCGTCGGCAGCGTCAGATGTGTATAAGAGACAG**CT**CCTACGGGAGGCAGCAG 3’

534R_ADA_2B

5’ GTCTCGTGGGCTCGGAGATGTGTATAAGAGACAG**CT**ATTACCGCGGCTGCTGGC 3’

1. 341F_ADA_3B

5’ TCGTCGGCAGCGTCAGATGTGTATAAGAGACAG**CAT**CCTACGGGAGGCAGCAG 3’

534R_ADA_3B

5’ GTCTCGTGGGCTCGGAGATGTGTATAAGAGACAG**ACT**ATTACCGCGGCTGCTGGC 3’

1. 341F_ADA_4B

5’ TCGTCGGCAGCGTCAGATGTGTATAAGAGACAG**TCAT**CCTACGGGAGGCAGCAG 3’

534R_ADA_4B

5’ GTCTCGTGGGCTCGGAGATGTGTATAAGAGACAG**CTAT**ATTACCGCGGCTGCTGGC 3’

1. 341F_ADA_5B

5’ TCGTCGGCAGCGTCAGATGTGTATAAGAGACAG**CTACT**CCTACGGGAGGCAGCAG 3’

534R_ADA_5B

5’ GTCTCGTGGGCTCGGAGATGTGTATAAGAGACAG**CATCT**ATTACCGCGGCTGCTGGC 3’

The optimized PCR conditions were: initial denaturation at 94 °C for 5 minutes, followed by 35 cycles of denaturation at 94 °C for 30 seconds, annealing at 69 °C for 30 seconds, extension at 72 °C for 30 seconds and a final extension cycle at 72 °C for 5 minutes. Paq5000 DNA polymerase (Agilent technologies, USA) was used and 5% DMSO was added to the master mix to enhance the concentration of amplified product from the metagenomic template.

The amplification of fungal ITS1 region was performed using Illumina Nextera XT adapter-ligated ITS1 region-specific primers, ITS1-ADA-F and ITS1-ADA-R ([Ihrmark et al., 2012](#_ENREF_2);[Tonge et al., 2014](#_ENREF_7)).

Primer sequences for amplification of fungal ITS1 region:

ITS1-ADA-F

5’ TCGTCGGCAGCGTCAGATGTGTATAAGAGACAGCTTGGTCATTTAGAGGAAGTAA 3’
ITS1-ADA-R

5’ GTCTCGTGGGCTCGGAGATGTGTATAAGAGACAGGCTGCGTTCTTCATCGATGC 3’

The optimized PCR conditions were: initial denaturation at 95 °C for 15 minutes (polymerase was added after this stage), followed by 35 cycles of denaturation at 94 °C for 1 minute, annealing at 66.5 °C for 2 minutes, extension at 72 °C for 2 minutes and a final extension cycle at 72 °C for 10 minutes. Paq5000 DNA polymerase (Agilent technologies, USA) was used and a final concentration of 2.5 mM MgCl_2_ was added to the PCR master mix to enhance the amplification of ITS1 region.

**Construction of Custom Database for ITS1 sequences**

For the construction of custom ITS1 database, the ITS1 reads generated after assembly were first assigned taxonomically using the ITS Database (verified and curated database downloaded from NCBI on Nov 30, 2015). The sequences which remained unassigned were aligned against NT database using blastn at NCBI web portal. The database sequences which aligned with the query were retrieved and were added to the previous database. All these sequences were clustered at ≥99% identity using UCLUST ([Edgar, 2010](#_ENREF_1)) and the representatives from these clusters were picked and were manually curated to create a custom reference database of ITS sequences. The database contained a total of 2,999 ITS sequences of different genera, of which six sequences belonged to uncultured species. This database was used as the reference for taxonomic assignment of ITS1 sequences.

**Gene quantification**

Gene quantification was carried out as described previously ([Qin et al., 2012](#_ENREF_5)). In brief, the high-quality reads were aligned against the combined gene catalogue using SOAP2 in SOAP aligner ([Li et al., 2009](#_ENREF_4)) for fungi and Bowtie2 for bacteria ([Langmead and Salzberg, 2012](#_ENREF_3)). For sequence-based profiling, two types of alignments were considered: (i) both pairs of a paired-end read completely map to a gene, or (ii) one end of the paired-end read maps to a gene and the other end remains unmapped. For both cases, the mapped read was counted as one copy. Further, the read count was normalized based on the length of the gene and was calculated as: $bi=\frac{xi}{Li}$

The relative abundance of a gene within the sample was calculated as:$ai=\frac{bi}{\sum j bj}=\frac{\frac{xi}{Li}}{\sum j\frac{xj}{Lj}}$

*a_i_*: relative abundance of gene 𝑖 in sample S; *x_i_*: the number of times a gene *i* can be detected in sample S (the number of mapped reads); *L_i_*: length of gene i; *b_i_*: copy number of gene *i* in sequenced data from sample S.

***Supplementary References***

Edgar, R.C. (2010). Search and clustering orders of magnitude faster than BLAST. *Bioinformatics* 26**,** 2460-2461.

Ihrmark, K., Bödeker, I.T., Cruz-Martinez, K., Friberg, H., Kubartova, A., Schenck, J., Strid, Y., Stenlid, J., Brandström-Durling, M., and Clemmensen, K.E. (2012). New primers to amplify the fungal ITS2 region–evaluation by 454-sequencing of artificial and natural communities. *FEMS microbiology ecology* 82**,** 666-677.

Langmead, B., and Salzberg, S.L. (2012). Fast gapped-read alignment with Bowtie 2. *Nat Methods* 9**,** 357-359.

Li, R., Yu, C., Li, Y., Lam, T.W., Yiu, S.M., Kristiansen, K., and Wang, J. (2009). SOAP2: an improved ultrafast tool for short read alignment. *Bioinformatics* 25**,** 1966-1967.

Qin, J., Li, Y., Cai, Z., Li, S., Zhu, J., Zhang, F., Liang, S., Zhang, W., Guan, Y., Shen, D., Peng, Y., Zhang, D., Jie, Z., Wu, W., Qin, Y., Xue, W., Li, J., Han, L., Lu, D., Wu, P., Dai, Y., Sun, X., Li, Z., Tang, A., Zhong, S., Li, X., Chen, W., Xu, R., Wang, M., Feng, Q., Gong, M., Yu, J., Zhang, Y., Zhang, M., Hansen, T., Sanchez, G., Raes, J., Falony, G., Okuda, S., Almeida, M., Lechatelier, E., Renault, P., Pons, N., Batto, J.M., Zhang, Z., Chen, H., Yang, R., Zheng, W., Yang, H., Wang, J., Ehrlich, S.D., Nielsen, R., Pedersen, O., and Kristiansen, K. (2012). A metagenome-wide association study of gut microbiota in type 2 diabetes. *Nature* 490**,** 55-60.

Soergel, D.A., Dey, N., Knight, R., and Brenner, S.E. (2012). Selection of primers for optimal taxonomic classification of environmental 16S rRNA gene sequences. *ISME J* 6**,** 1440-1444.

Tonge, D.P., Pashley, C.H., and Gant, T.W. (2014). Amplicon–Based Metagenomic Analysis of Mixed Fungal Samples Using Proton Release Amplicon Sequencing. *PLoS One* 9**,** e93849.

Wang, X.C., Liu, C., Huang, L., Bengtsson‐Palme, J., Chen, H., Zhang, J.H., Cai, D., and Li, J.Q. (2015). ITS1: a DNA barcode better than ITS2 in eukaryotes? *Molecular ecology resources* 15**,** 573-586.

Wang, Y., and Qian, P.Y. (2009). Conservative fragments in bacterial 16S rRNA genes and primer design for 16S ribosomal DNA amplicons in metagenomic studies. *PLoS One* 4**,** e7401.
