# Supplementary material for: Comparison of Healthy and Dandruff Scalp Microbiome Reveals the Role of Commensals in Scalp Health
Source: Front Cell Infect Microbiol. 2018 Oct 4;8:346. doi: 10.3389/fcimb.2018.00346 (PMC6180232; doi:10.3389/fcimb.2018.00346)
Supplement: Supplementary file 2 [file Table_1.docx]

**Table S1. Demography**

| Demographics | *n* | Mean (SD) | Median | Min;Max |
| --- | --- | --- | --- | --- |
| Age | 140 | 34.6 (6.4) | 35.0 | 20.0;45.0 |
| Height (cm) | 140 | 154.2 (6.6) | 154.0 | 137.0;182.0 |
| Weight (Kg) | 140 | 65.2 (14.5) | 62.9 | 35.1;115.5 |
| BMI (kg/m²) | 140 | 27.3 (5.4) | 26.6 | 14.1;44.7 |
