# Supplementary material for: Comparison of Healthy and Dandruff Scalp Microbiome Reveals the Role of Commensals in Scalp Health
Source: Front Cell Infect Microbiol. 2018 Oct 4;8:346. doi: 10.3389/fcimb.2018.00346 (PMC6180232; doi:10.3389/fcimb.2018.00346)
Supplement: Supplementary file 4 [file Table_3.docx]

**Table S3. Genomic DNA isolation of individual bacterial and fungal species.** List of the strains used to establish the DNA extraction method and the genomic DNA amount obtained for each species.

|  | **Species** | **Strain*** | **Number of cells** | **Genomic DNA (ng)** |
| --- | --- | --- | --- | --- |
| Bacteria | *Propionibacterium acnes* | CIPA179^a^ | 1 x 10^8^ | 175 ± 10 |
|  | *Staphylococcus capitis* | CIP81.53T^a^ | 1 x 10^8^ | 190 ± 20 |
|  | *Staphylococcus caprae* | CIP104000^a^ | 1 x 10^8^ | 77 ± 6 |
|  | *Staphylococcus epidermidis* | Clavaud *et al*. 2013 | 1 x 10^8^ | 199 ± 11 |
|  | *Corynebacterium mucifaciens* | CIP105129^a^ | 1 x 10^8^ | 64 ± 12 |
|  | *Corynebacterium xerosis* | CIP5216 ^a^ | 1 x 10^8^ | 85 ± 9 |
|  | *Corynebacterium striatum* | CIP 8115 ^a^ | 1 x 10^8^ | 161 ± 32 |
|  |  |  |  |  |
| Fungi | *Candida albicans* | ATCC10231 ^b^ | 1 x 10^8^ | 60 ± 4 |
|  | *Malassezia restricta* | CBS7877^b^ | 1 x 10^8^ | 86 ± 23 |
|  | *Malassezia globosa* | CBS7874^b^ | 1 x 10^8^ | 90 ± 8 |

*Strains were cultivated as recommended by the provider library: ^a^Biological Resource Center of Institut Pasteur (Institut Pasteur, Paris, France); ^b^Centraal bureau voor Schimmelcultures (CBS, Utrecht, Netherlands).

In addition, the two DNA extraction methods were tested combinedly for the three-major species: *S. epidermidis*, *P. acnes* and *M. restricta*. The ‘bacterial extraction method’ applied to M. restricta lead to only 3.31 ± 0.31 ng of the total DNA (compared to 86 ± 23 ng obtained using the fungal method). Further, the ‘fungal extraction method’ applied to S. epidermidis and P. acnes gave 4.55 ± 1.12 ng and 30.82 ± 8.78 ng of the total DNA, respectively (compared to 199 ± 11 ng and 175 ± 10 obtained using the bacterial method).
